# Modelling behavioural changes and vaccination in the transmission of respiratory viruses with co–infection

**DOI:** 10.1007/s00285-025-02280-3

**Published:** 2025-09-16

**Authors:** Bruno Buonomo, Emanuela Penitente

**Affiliations:** Department of Mathematics and Applications, University of Naples Federico II, via Cintia, I-80126 Naples, Italy

**Keywords:** Mathematical epidemiology, Respiratory viruses, Co–infection, Information, Human behaviour, 92D30, 34C60

## Abstract

We consider a mathematical model to explore the effects of human behavioural changes on the transmission of two respiratory viruses, where co–infection is possible. The model includes an index to describe the human choices induced by information and rumours regarding the diseases. We first consider the case in which the public health authorities rely only on non–pharmaceutical containment measures and perform a qualitative analysis of the model through bifurcation theory, in order to analyse the existence and stability of both endemic and co–endemic equilibria. We also show the impact of the most relevant information–related parameters on the system dynamics. Then, we extend the model by assuming that a vaccine is available for each of the two viruses. We show how adherence to social distancing may be affected by information and rumours regarding the vaccination coverage in the community. Finally, we investigate the effects of seasonality by introducing a two–state switch function to represent a reduction in both vaccination and transmission rates during the summer season. We found that seasonality causes an increase in the prevalence peaks, suggesting that the detrimental effects due to the reduction of vaccination rates prevail over the beneficial ones due to the reduction of transmission.

## Introduction

A co–infection between two respiratory viruses is defined as the simultaneous infection of an individual with two genetically different viruses that infect the respiratory system (Cox 2001). This phenomenon can involve influenza or parainfluenza viruses, coronaviruses (e.g., MERS–CoV, SARS–CoV–2), adenoviruses, respiratory syncytial virus (RSV) and human rhinoviruses (HRV). Co–infection can occur between viruses belonging to different species or also between different strains of the same virus (Cox 2001). The interaction between two different viruses can be classified into two categories: positive interaction, when the two viruses act together worsening the symptoms and outcome of the disease, and negative interaction, when the presence of one virus inhibits the replication of the other virus in the host’s body (Devi et al. 2021). Implications of positive interactions between Influenza A virus (IAV) and SARS–CoV–2 include enhanced host infectivity (Bai et al. 2021) as well as greater body weight loss and more severe lung damage in co–infected hamsters (Kinoshita et al. 2021). As for negative interactions, one of the most common mechanisms of negative viral-viral interaction is viral interference, which occurs when the presence of one virus (the interfering one) in a host inhibits the replication or activity of another virus (Wu et al. 2020). Several couples of respiratory viruses have this particular feature, e.g., HRV and IAV, HRV and RSV, HRV and SARS–CoV–2 (Piret and Boivin 2022).

Another relevant aspect about co–infections is that when two different viruses or two different strains of the same virus infect a host, they can gain new properties through phenomena like *recombination* and *reassortment* (Bolze et al. 2022; Zhang and Cao 2014). Recombination is defined as the exchange of genetic segments between two viral genomes which co-infect the same host cell. Reassortment is a particular type of recombination and concerns viruses with segmented genomes. It occurs when the two viruses swap complete genome segments, giving rise to new segment combinations (Pérez-Losada et al. 2015).

Since the onset of the COVID–19 pandemic, several studies have investigated the frequency of co–infections with SARS–CoV–2 (Dadashi et al. 2021; Krumbein et al. 2023; Dao et al. 2021; Yan et al. 2023). The proportion of co–infected individuals can vary significantly depending on several factors including the season, the geographical region, age group, sex and health status. Dadashi et al. (2021) performed a systematic review of papers published from December 2019 to September 2020, finding an overall influenza prevalence of 0.8% in confirmed cases of COVID–19. In 2023, Krumbein et al. (2023) conducted a meta–analysis to determine the proportion of co–infections with any respiratory virus between SARS–CoV–2 patients to identify the most frequent co-pathogens and to compare the clinical outcomes. They found that the most common co-pathogens are influenza virus (1.54%) and enteroviruses (1.32%). Furthermore, co–infected patients are significantly more exposed to the risk of death than patients infected only with SARS–CoV–2. Similar estimates were obtained by Dao et al. (2021) and Yan et al. (2023). The latter study also shows that co–infected have a higher probability of developing severe outcomes when compared with mono–infected patients. In addition, the co–infection proportion is significantly high in dead patients (36.67%).

Overall, although the proportion of co–infections is generally low, co–infections may have a relevant impact on the course of epidemic outbreaks in terms of both the disease burden and their potential ability to generate new variants or entirely new viruses with unknown impact on transmissibility and disease severity (Bolze et al. 2022). Consequently, it is essential to conduct further research into the underlying mechanisms of the phenomenon and the possible consequences of co–infections on the population. Mathematical modelling may provide useful tools to assess the dynamics of respiratory virus co–infection and to provide information to public health practitioners regarding the implementation of pharmaceutical and non–pharmaceutical mitigation strategies (Majeed et al. 2023; Fahlena et al. 2022).

In the last few years, and especially after the COVID–19 global pandemic, mathematical models designed to investigate respiratory viruses co–infection dynamics began to catch the attention of scholars. Several intriguing models and analyses can be found in the recent literature. In 2013, Zhang et al. developed a SIR (Susceptible–Infectious–Removed) compartmental model including two strains of influenza virus (Zhang et al. 2013). Their model takes into account co–infection as well as reassortment. Pinky et al. proposed a co–infection model where an influenza–like virus blocks infection with SARS–CoV–2 (Pinky and Dobrovolny 2022). More recently, Bhowmick et al. considered SARS–CoV–2 and influenza–like viruses (ILI) co–infection and incorporated the saturated treatment rate to evaluate the impact of limited treatment resources on the time evolution of the epidemic (Bhowmick et al. 2023). In their model, it is assumed that direct co–infection is possible, that is susceptible individuals may become infected simultaneously with SARS–CoV–2 and ILI. However, there is no evidence that direct co–infection is particularly likely to occur, therefore it is usually neglected when modelling co–infections (Hamelin et al. 2019). Direct co–infection is not considered by Fahlena et al. (2022), who introduced a SIR–like compartmental model designed to describe the interaction of two generic respiratory viruses. Their model has the advantage of being detailed, mathematically tractable and sufficiently general to be applied both in the case of positive and negative interaction between respiratory viruses.

All the aforementioned models can provide useful insights into the co–infection dynamics. However, they do not consider an important aspect, namely the human behavioural changes and their effects on the course of the epidemic. Indeed, it is widely acknowledged that human behaviour is a key determinant of the effectiveness of mitigation strategies during an epidemic, especially when the adoption of self–protective measures is based essentially on free will (Bergstrom and Hanage 2024; Lewnard and Lo 2020; West et al. 2020). Information and rumours about the spread of the disease may be a critical factor as to whether or not the individuals adopt protective measures, like social distancing, wearing masks, vaccination, and so on (Collinson et al. 2015; Wang et al. 2016). During the recent COVID–19 pandemic, it has been clear that social distancing, together with vaccines and pharmacological treatments is essential to mitigate the disease (Khataee et al. 2021; Zhou et al. 2020). However, in some cases, people did not adhere to these measures due to various reasons, such as the perceived high risk of vaccine side effects or the perceived low risk of infection due to low prevalence or high vaccination coverage. These behaviours have posed additional problems for the public health systems (Masters et al. 2020; Troiano and Nardi 2021; Dror et al. 2020). This example underscores the crucial role of information–dependent behavioural changes in both mitigating and fuelling epidemics.

Motivated by the above–mentioned considerations, we consider in this paper a mathematical model for co–infections of respiratory viruses that takes into account information–dependent human behavioural changes. We use the SIR–like model proposed by Fahlena et al. (2022) as baseline model and augment it with the *information index*, a state variable defined by a distributed–delay equation. Such an index was introduced in 2007 by A. d’Onofrio and P. Manfredi to describe the information–induced perception regarding the status of the disease (Manfredi and d’Onofrio 2013; d’Onofrio et al. 2007) and is nowadays a consolidated tool in the field of Behavioural Epidemiology of Infectious Diseases (BEID) (Manfredi and d’Onofrio 2013; Wang et al. 2016).

Firstly, we consider the case in which the public health authorities rely only on non–pharmaceutical containment measures that reduce contact patterns (e.g., social distancing). Assuming an exponentially fading memory kernel, we derive a system of non–linear ordinary differential equations. We conduct a qualitative analysis using stability and bifurcation theory, emphasizing both the role of the control reproduction number, $\mathcal {R}_C$, and the invasion numbers of the two viruses. In particular, we show that the disease–free equilibrium destabilises at $\mathcal {R}_C=1$ via a transcritical forward bifurcation; in addition, we give sufficient conditions for the existence of locally stable co–endemic equilibria by proving the existence of a forward bifurcation when the minimum between the two invasion numbers crosses the threshold value of 1. Through numerical simulations, we also show the impact of the most relevant information–related parameters on the system dynamics.

Secondly, we extend the model by assuming that a vaccine is available for each of the two viruses. For each of them, the vaccination choice is assumed to be partially voluntary and dependent on information about the spread of the disease in both the present and recent past. We show how adherence to social distancing may be affected by the information and rumours concerning the vaccination coverage in the community.

Finally, we numerically show the effects of seasonality on the course of epidemics. In fact, many respiratory viral diseases exhibit a seasonal pattern, with outbreaks often peaking during certain times of the year (Moriyama et al. 2020). The seasonality may be caused by several factors, including environmental conditions and human behaviour (Neumann and Kawaoka 2022). We include seasonality in the model by considering reduced vaccination and transmission rates in the summer season.

The rest of the paper is organized as follows: the model is introduced in Section 2. In Section 3, the control reproduction numbers and the invasion numbers are computed. Moreover, the existence and stability of the equilibria are established, and the occurrence of transcritical bifurcations is shown. In Section 4, we investigate through numerical simulations how human behavioural changes affect the dynamics of the model. In Section 5, we generalise the model by considering both information–induced vaccination and vaccination–induced relaxation of non–pharmaceutical containment measures. We discuss the main results and give an outlook on possible research perspectives in Section 6.

**Fig. 1:**
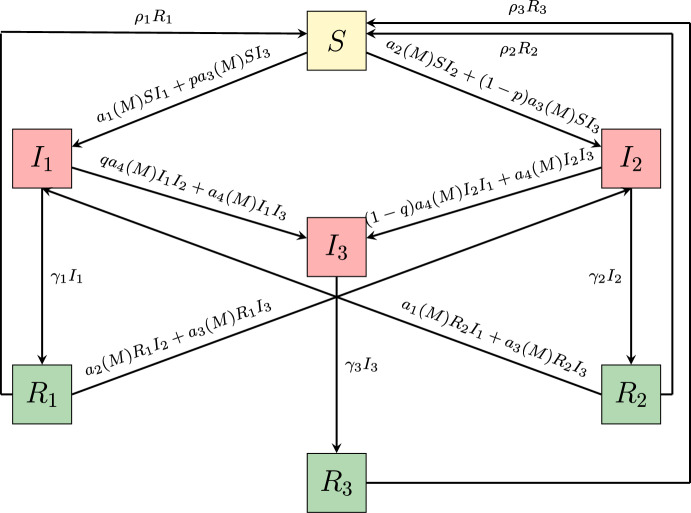
Flowchart of the model (6). The recruitment of susceptibles and the natural death of individuals in each compartment have not been reported

## The model

### State variables and the information index

Let us consider two generic respiratory viruses, named virus–1 and virus–2. We assume that the total population is divided into seven disjointed compartments: susceptible (*S*), infected only by virus–1 ($$I_1$$), infected only by virus–2 ($$I_2$$), co–infected ($$I_3$$), recovered only from virus–1 ($$R_1$$), recovered only from virus–2 ($$R_2)$$ and recovered from co–infection ($$R_3$$). The size of each compartment at time *t* represents a state variable of the model. The size of the total population at time *t*, denoted by *N*(*t*), is given by:

$$\begin{aligned} N(t) = S(t) + \sum _{i=1}^{3}I_i(t) + \sum _{i=1}^{3}R_i(t). \end{aligned}$$

In order to model the effects of information–dependent behavioural changes, we assume that the individual choice to use disease–protective devices is partially voluntary and depends on the available information about the spread of the disease in the community. Information is mathematically modelled by the *information index* *M*(*t*), which contains information about the current and past values of the diseases and is defined as the follows (d’Onofrio et al. 2007; d’Onofrio and Manfredi 2009):

1 $$\begin{aligned} M(t) = \int _{0}^{+\infty } g \big ( I_1(t- \tau ), I_2(t- \tau ), I_3(t- \tau ) \big ) \text {Erl}_{1,a}(\tau ) \, d\tau . \end{aligned}$$

In the latter expression, *g* is called *message* function and $\hbox {Erl}_{1,a}$ denotes the *first–order Erlang* distribution kernel (also known as *exponentially fading* kernel), i.e.:

2 $$\begin{aligned} \text {Erl}_{1,a}(\tau )= ae^{-a \tau }, \qquad \tau \in \mathbb {R}^+, \end{aligned}$$

where the constant $a\in \mathbb {R}^+$ is the *rate* parameter of the distribution.

As for the message function *g*, it describes the information that influences the individual’s choice to adopt or not to adopt self–protective measures. We consider the following function:

3 $$\begin{aligned} g(I_1, I_2, I_3) = k_1 I_1 + k_2 I_2 + k_3I_3. \end{aligned}$$

Here, the term $k_iI_i$, for $i=1,2,3,$ defines the perceived risk of infection, which is assumed to be proportional to the infection prevalence $I_i$ (Wang et al. 2016). The parameter $k_i\in [0,1]$, for $i=1,2,3,$ is the *information coverage* regarding the infected by virus–1, virus–2 and both viruses, respectively. Such parameter represents the balance between two different processes: the disease under-reporting – here assumed to be the prevailing process – due mainly to routine procedures, and the overestimation usually induced by media and rumours on the disease status (Buonomo et al. 2008).

### Modelling the transmission rates

Let us denote by $a_1$ (resp. $a_2$) the transmission rate of virus–1 (resp. virus–2), and by $\pi _1$ (resp. $\pi _2$) the probability of successful infection after contact with an individual infected by virus–1 (resp. virus–2). Moreover, denote by $a_3$ the rate at which co–infected individuals transmit one of the two viruses and let $\pi _3$ be the corresponding probability of successful infection. Finally, denote by $a_4$ the *co–infection rate*, i.e., the rate at which the individuals infected from one virus also contract the other virus and let $\pi _4$ be the corresponding probability of successful infection.

We assume that the public health system enacts campaigns to encourage the reduction of interpersonal contacts (e.g., social distancing, wearing masks, etc.). The individual’s compliance with the recommendations is assumed to depend partially on the information about the spread of the disease in both the present and recent past. Since every transmission rate can be seen as the product of the contact rate times the probability of getting infected after contact with an infectious individual, we assume that the contact rate depends on the information index *M*(*t*). We set:

4 $$\begin{aligned} a_j(M) = \pi _j[\hat{c} - c_0 - c_1(M)], \qquad j =1, \dots , 4 , \end{aligned}$$

where $\hat{c}$ is the “baseline” contact rate (i.e., the contact rate when no reduction strategy is in place), $c_0$ is the information–independent reduction rate due to compliance with public health policies and $c_1(M)$ is the rate of reduction in interpersonal contacts due to information–dependent voluntary compliance with governmental policies. The function $c_1$ is assumed to be increasing with *M*, $c_1(0)=0$, and $\hat{c} > c_0 + \text {max}(c_1(M))$. The last inequality guarantees that $a_j(M)$ is positive for all values of *M*.

Equation (4) may be rewritten as:

5 $$\begin{aligned} a_j(M) = \hat{a}_j - a_{j,0} - a_{j,1}(M), \end{aligned}$$

where $\hat{a}_j = \pi _j \hat{c}$, $a_{j,0} = \pi _j c_0$ and $a_{j,1}(M) = \pi _j c_1(M)$. A specific form of $a_{j,1}(M)$ will be given in subsection 2.4.

### The balance equations

The balance equations for the seven compartments *S*(*t*), $I_i(t)$ and $R_i(t)$, for $i=1,2,3$ are illustrated in the flowchart in Figure 1. As mentioned above, the model structure follows the framework introduced by Fahlena et al. (2022), in which disease-induced mortality is neglected and the total population size is assumed to be constantly equal to its initial value. In our formulation, we extend this model by introducing the human behavioural changes through the information index (1) – (3) and the information–dependent rates $a_1, a_2, a_3$ and $a_4$ defined in (5). We get the following system of non–linear ordinary differential equations:

6a $$\begin{aligned} \dot{S}(t)&= \mu - a_1(M)SI_1 -a_2(M)SI_2 - a_3(M)SI_3 + \rho _1R_1 \nonumber \\&\quad + \rho _2R_2 + \rho _3R_3 -\mu S,\end{aligned}$$

6b $$\begin{aligned} \dot{I_1}(t)&= a_1(M)SI_1 + pa_3(M)SI_3 -\gamma _1I_1 + a_1(M)R_2I_1 \nonumber \\&\quad + a_3(M)R_2I_3- qa_4(M)I_1I_2 - a_4(M)I_1I_3 - \mu I_1, \end{aligned}$$

6c $$\begin{aligned} \dot{I}_2(t)&= a_2(M)SI_2 + (1-p)a_3(M)SI_3 - \gamma _2I_2 + a_2(M)R_1I_2 \nonumber \\&\quad + a_3(M)R_1I_3 - (1-q)a_4(M)I_2I_1 - a_4(M)I_2I_3 - \mu I_2,\end{aligned}$$

6d $$\begin{aligned} \dot{I}_3(t)&= a_4(M)I_1I_2 + a_4(M)I_1I_3 + a_4(M)I_2I_3 - \gamma _3I_3 - \mu I_3, \end{aligned}$$

6e $$\begin{aligned} \dot{R}_1(t)&= \gamma _1I_1 - a_2(M)R_1I_2 - a_3(M)R_1I_3 - \rho _1 R_1 - \mu R_1,\end{aligned}$$

6f $$\begin{aligned} \dot{R}_2(t)&= \gamma _2I_2 - a_1(M)R_2I_1 - a_3(M)R_2I_3 - \rho _2R_2 - \mu R_2,\end{aligned}$$

6g $$\begin{aligned} \dot{R}_3(t)&= \gamma _3I_3 - \rho _3R_3 - \mu R_3,\end{aligned}$$

6h $$\begin{aligned} \dot{M}(t)&= \frac{1}{T_a}(k_1I_1 +k_2I_2 +k_3I_3 - M), \end{aligned}$$

where the upper dots denote the time derivative. Equation (6h) is obtained by time derivative of the information index (1) with (2) – (3) and $T_a=a^{-1}$ is the characteristic memory length, which can be interpreted as the average time delay in the collection of information of the disease (Wang et al. 2016).

The system (6) is equipped with initial conditions to set up a Cauchy problem:

7 $$\begin{aligned} S(t_0) > 0, \quad I_i(t_0) \ge 0, \quad R_i(t_0) \ge 0, \quad \text {for } i =1,2,3. \end{aligned}$$

In our setting, we normalise the initial population size by setting $N(t_0) = 1$, so that $N(t) = 1$ for $t > t_0$. Finally, system (6) – (7) can be rewritten in the vectorial form:

8 $$\begin{aligned} \dot{\textbf{x}}(t) = \textbf{F}\bigl (\textbf{x}(t)\bigr ), \qquad \textbf{x}(t_0) = \textbf{x}_0, \end{aligned}$$

where $\textbf{x}(t) = (x_i(t))_{i=1, \dots , 8} = (S(t), I_1(t), I_2(t), I_3(t), R_1(t), R_2(t), R_3(t), M(t))$, $\textbf{F}(\textbf{x}) = (f_1(\textbf{x}), f_2(\textbf{x})$, $f_3(\textbf{x}), f_4(\textbf{x}), f_5(\textbf{x}), f_6(\textbf{x}), f_7(\textbf{x}), f_8(\textbf{x}))$ is the autonomous vector field and $\textbf{x}_0$ satisfies the inequalities (7).

### Functional forms of the information–dependent transmission rates

We specify the functional form of the contact rate $c_1(M)$ in equation (4) that will be used for all the numerical examples. As mentioned in subsection 2.2, the contact rate $c_1(M)$ is assumed to be increasing with *M*, $c_1(0) = 0$ and max$(c_1(M))< \hat{c} - c_0$. These properties are satisfied by a Holling type II functional:

$$\begin{aligned} c_1(M) = (\hat{c}- c_0) \frac{\delta M}{1 + \delta M}, \end{aligned}$$

where $\delta$ is a positive constant tuning the reactivity of the population to adopt social distancing. This type of functional response has been extensively adopted to represent the information–dependent part of the contact rate (see, e.g., d’Onofrio and Manfredi 2009; Buonomo and Della Marca 2020). With this choice, the transmission rate $a_j(M)$ in equation (5) assumes the form:

9 $$\begin{aligned} a_j(M) = \frac{\hat{a}_j- a_{j,0}}{1 + \delta M}, \end{aligned}$$

for $$j = 1, \dots , 4$$.

## Basic properties and equilibria

### Biologically feasible region

#### Proposition 1

Every solution of system (8) exists, is unique in the interval $[t_0, \infty )$ and is contained in the positively invariant set $\Omega$ defined by:

10 $$\begin{aligned} \Omega = \left\{ \textbf{x} \in \mathbb {R}_+^8 \, : \, \sum _{i=1}^7 x_i =1, \, \,\, x_8 \le \sum _{i=1}^{3} k_i \right\} , \end{aligned}$$

where $\mathbb {R}_+^8$ is the non–negative orthant of $\mathbb {R}^8$.

#### Proof

See Appendix A. $$\square $$

### Reproduction numbers and disease extinction

Two key quantities related to model (6) are the basic reproduction number $\mathcal {R}_0$ and the control reproduction number $\mathcal {R}_C$. As it is well–known, the former is the average number of secondary cases produced by an infected individual in a fully susceptible population over the full course of the infectious period (Diekmann et al. 1990). The latter has the same meaning, but in the presence of strategies to contain the epidemic (Gumel et al. 2004). Therefore, $\mathcal {R}_0$ may be obtained directly from the baseline non–behavioural model proposed by Fahlena et al. (2022). They found that:

11 $$\begin{aligned} \mathcal {R}_0 = \max \left\{ \mathcal {R}_{01}, \mathcal {R}_{02} \right\} , \end{aligned}$$

where

12 $$\begin{aligned} \mathcal {R}_{01} = \frac{\hat{a}_1}{\mu + \gamma _1} , \qquad \mathcal {R}_{02} = \frac{\hat{a}_2}{\mu + \gamma _2}. \end{aligned}$$

As for the control reproduction number, once observed that model (6) admits the disease–free equilibrium

$$\begin{aligned} E_0 = (1, 0, 0, 0, 0, 0, 0, 0), \end{aligned}$$

we can state the following proposition:

#### Proposition 2

The control reproduction number $\mathcal {R}_C$ for model (6) is given by

13 $$\begin{aligned} \mathcal {R}_C = \left( 1- \frac{c_0}{\hat{c}} \right) \, \mathcal {R}_0. \end{aligned}$$

#### Proof

We follow the notation adopted by Van den Driessche and Watmough (2002) and introduce:

$$\begin{aligned} \mathcal {F} = { \begin{bmatrix} a_1(M)S I_1 + pa_3(M)SI_3 + a_1(M)R_2I_1 + a_3(M)R_2I_3\\ a_2(M)SI_2 + (1-p)a_3(M)SI_3 +a_2(M)R_1I_2 + a_3(M)R_1I_3\\ a_4(M)I_1I_2 + a_4(M)I_1I_3 + a_4(M)I_2I_3 \end{bmatrix}} \end{aligned}$$

and

$$\begin{aligned} { \mathcal {V} = \begin{bmatrix} qa_4(M)I_1I_2 + a_4(M)I_1I_3+ (\gamma _1+ \mu )I_1\\ (1-q)a_4(M)I_2I_1 + a_4(M)I_2I_3 + (\gamma _2+ \mu )I_2\\ (\gamma _3+ \mu )I_3 \end{bmatrix}.} \end{aligned}$$

The Jacobian of $\mathcal {F}$ and $\mathcal {V}$ evaluated at the disease–free equilibrium $E_0$ are the so–called *transmission* matrix *F* and the *transition* matrix *V* (Diekmann et al. 2010):

$$\begin{aligned} { F = \begin{bmatrix} a_1(0) & \quad 0 & \quad pa_3(0)\\ 0 & \quad a_2(0) & \quad (1-p)a_3(0)\\ 0 & \quad 0 & \quad 0 \end{bmatrix}, } \end{aligned}$$

and

$$\begin{aligned} { V = \begin{bmatrix} \mu + \gamma _1 & \quad 0 & \quad 0\\ 0 & \quad \mu + \gamma _2 & \quad 0\\ 0 & \quad 0 & \quad \mu + \gamma _3 \end{bmatrix}. } \end{aligned}$$

The control reproduction number $\mathcal {R}_C$ is the spectral radius of the next generation matrix (NGM), usually denoted by *K* and given by $K = FV^{-1}$. We have:

$$\begin{aligned} { K = \begin{bmatrix} \frac{a_1(0)}{\mu + \gamma _1} & \quad 0 & \quad \frac{pa_3(0)}{\mu + \gamma _3}\\ 0 & \quad \frac{a_2(0)}{\mu + \gamma _2} & \quad \frac{(1-p)a_3(0)}{\mu + \gamma _3}\\ 0 & \quad 0 & \quad 0 \end{bmatrix}. } \end{aligned}$$

By denoting

14 $$\begin{aligned} \mathcal {R}_{C1} = \frac{a_1(0)}{\mu + \gamma _1} = \frac{\hat{a}_1- a_{1,0}}{\mu + \gamma _1}, \qquad \mathcal {R}_{C2}= \frac{a_2(0)}{\mu + \gamma _2} = \frac{\hat{a}_2- a_{2,0}}{\mu + \gamma _2} , \end{aligned}$$

we finally get

$$\begin{aligned} \mathcal {R}_C&= \text {max} \left\{ \mathcal {R}_{C1},\mathcal {R}_{C2} \right\} = \\&= \text {max} \left\{ \mathcal {R}_{01}\left( 1- \frac{c_0}{ \hat{c}} \right) \, , \, \mathcal {R}_{02}\left( 1- \frac{c_0}{\hat{c}} \right) \, \right\} \,\\&= \left( 1-\frac{c_0}{\hat{c}} \right) \mathcal {R}_0. \end{aligned}$$

$$\square $$

**Fig. 2:**
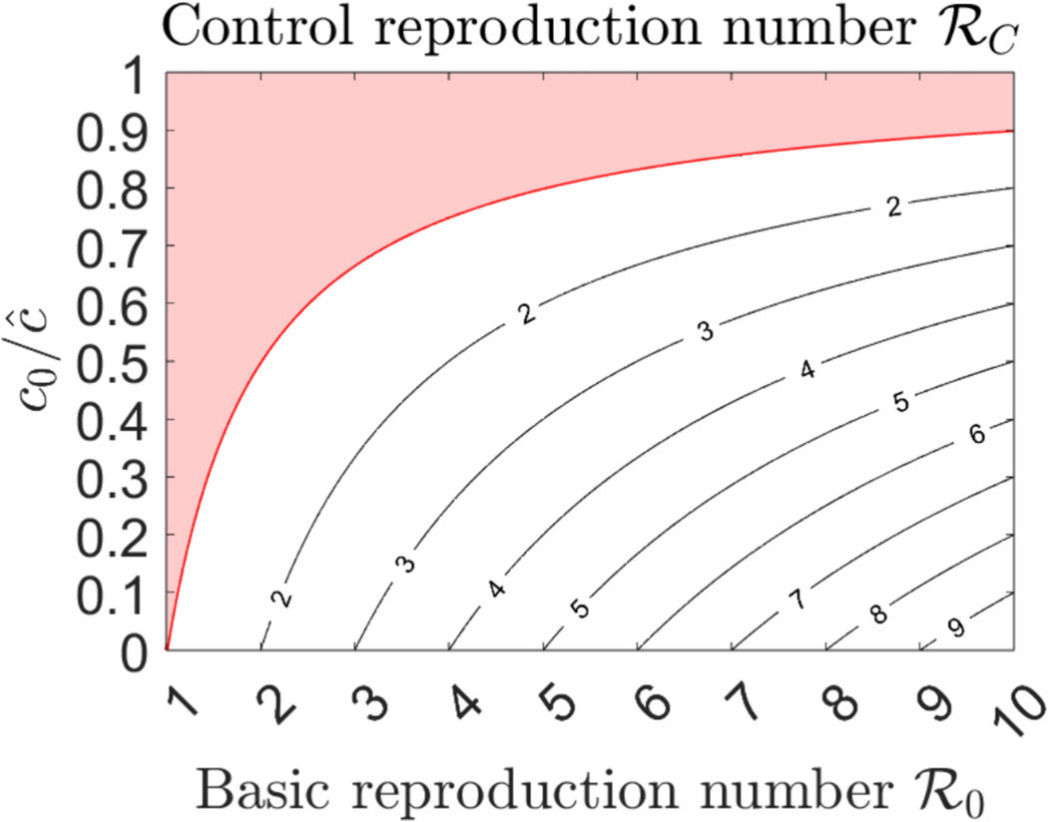
Contour plot of the control reproduction number $\mathcal {R}_C$ versus the basic reproduction number $\mathcal {R}_0$ and the ratio $c_0/\hat{c}$. The light–red coloured region satisfies $\mathcal {R}_C < 1$, the red line represents the curve $c_0/\hat{c} =1- 1/\mathcal {R}_0$

#### Proposition 3

If $\mathcal {R}_C < 1$, the disease-free equilibrium $E_0$ of model (6) is locally asymptotically stable. If $\mathcal {R}_C > 1$, then $E_0$ is unstable.

#### Proof

It is straightforward to show that the eigenvalues of the Jacobian matrix $J(E_0)$ are all real negative eigenvalues except for $\lambda _1 = (\gamma _1 + \mu )( \mathcal {R}_{C1}-1)$ and $\lambda _2 = (\gamma _2 + \mu )( \mathcal {R}_{C2}-1)$. $\square$

#### Remark 1

(i) Due to the stability result obtained in Proposition 3, from a public policy perspective, the information–independent reduction rate due to compliance to public health policies, $c_0$, must be such that $c_0> \hat{c}(1-1/\mathcal {R}_0)$ to ensure that $\mathcal {R}_C <1$. The contour lines $c_0/\hat{c}$ versus $\mathcal {R}_0$ are shown in Figure 2. The red line represents the curve $c_0/\hat{c} = 1/\mathcal {R}_0$, and the light–red region corresponds to parameter values that guarantee the elimination of both viruses.

(*ii*): The quantities $\mathcal {R}_{Ci}$ are the control reproduction numbers of virus–*i* $i=1,2$, i.e., the average number of secondary cases of virus–*i* produced by a single individual infected with virus–*i*, in a population fully susceptible to both viruses, during the entire duration of the infectious period, in presence of control measures.

### Boundary equilibria and invasion criteria

The model admits a unique virus–*i*–dominance equilibrium $E_i$ ($i=1,2$), a boundary equilibrium that exists if and only if $\mathcal {R}_{Ci} > 1$ and represents a steady state in which only virus–*i* is endemic in the population. Precisely, we have:

$$\begin{aligned} E_1 = (S_1^*, I_1^*, 0, 0, R_1^*, 0, 0, M_1^*), \qquad \end{aligned}$$

where:

$$\begin{aligned} S_1^* = \frac{\mu + \gamma _1}{a_1(g(I_1^*))}, \quad R_1^* = \frac{\gamma _1}{\mu + \rho _1}I_1^*, \quad M_1^*= k_1 I_1^*, \end{aligned}$$

and $I_1^*$ is the unique positive root of the function

15 $$\begin{aligned} \zeta (I_1) = \mu \left[ 1- \frac{\mu + \gamma _1 }{a_1(g(I_1))}\right] + I_1\left[ \frac{\rho _1 \gamma _1}{\mu + \rho _1}- (\gamma _1+\mu )\right] , \end{aligned}$$

i.e., the coordinate $I_1^*$ satisfies

$$\begin{aligned} { I_1^* = \frac{(\mu + \rho _1)(a_1^*-\mu -\gamma _1)}{(\mu +\rho _1 + \gamma _1)a_1^*}.} \end{aligned}$$

Since $\zeta$ is monotonically decreasing in $I_1$ and $\zeta (0) = \mu (1-1/\mathcal {R}_{C1})$, there exists a unique positive root $I_1^*$ if and only if $\mathcal {R}_{C1} > 1$.

Analogously, the virus–2–dominance equilibrium $E_2=(S_2^*, 0, I_2^{*},0,0,R_2^{*},0,M_2^{*})$ exists and is unique if and only if $\mathcal {R}_{C2} > 1$.

In order to study the local stability of the virus–*i* dominance equilibrium, we compute the invasion number $\mathcal {R}_j^{\text {inv}}$ of virus–*j* at equilibrium $E_i$. It is defined as the number of secondary infections that one individual infected with virus–*j* will produce in a population in which virus–*i* is endemic, during its infectivity period (Zhang et al. 2007; Mitchell and Kribs 2019). Using the method developed by Van den Driessche and Watmough (2002), we obtain the following expression for $\mathcal {R}_2^{\text {inv}}$:

16 $$\begin{aligned} \mathcal {R}_2^{\text {inv}} = \frac{\Pi _{11}+\Pi _{22} + \sqrt{(\Pi _{11}-\Pi _{22})^2 + 4\Pi _{12}\Pi _{21}}}{2}, \end{aligned}$$

where:

$$\begin{aligned} \Pi _{11}&= \frac{[(\mu + \rho _1)(\mu + \gamma _1) + \gamma _1a_1^*I_1^*]a_2^*}{(\mu +\rho _1)[(1-q) a_4^*I_1^*+ \mu + \gamma _2]a_1^*},&\Pi _{22}&= \frac{a_4^*I_1^*}{\mu + \gamma _3},\\ \Pi _{12}&= \frac{[(1-p)(\mu + \rho _1)(\mu + \gamma _1)+ \gamma _1 a_1^*I_1^*]a_3^*}{(\mu + \rho _1)(\mu + \gamma _3)a_1^*},&\Pi _{21}&= \frac{a_4^*I_1^*}{(1-q) a_4^*I_1^* + \mu + \gamma _2}, \end{aligned}$$

and $a_i^*=a_i(g(I_1^*))$ is the value of the *i*–th transmission rate ($i=1, \dots , 4$) at the equilibrium $E_1$. Further details on the computation of $\mathcal {R}_2^{\text {inv}}$ are contained in Appendix B.

Analogously, the invasion number $\mathcal {R}_1^{\text {inv}}$ of virus–1 at the equilibrium $E_2$ has the following expression:

17 $$\begin{aligned} \mathcal {R}_1^{\text {inv}} = \frac{\Sigma _{11}+\Sigma _{22} + \sqrt{(\Sigma _{11}-\Sigma _{22})^2 + 4\Sigma _{12}\Sigma _{21}}}{2}, \end{aligned}$$

where:

$$\begin{aligned} \Sigma _{11}&= \frac{[(\mu + \rho _2)(\mu + \gamma _2) + \gamma _2a_2^*I_2^{*}]a_1^{*}}{(\mu +\rho _2)[q a_4^{*}I_2^{*}+ \mu + \gamma _1]a_2^{*}},&\Sigma _{22}&= \frac{a_4^{*}I_2^{*}}{\mu + \gamma _3},\\ \Sigma _{12}&= \frac{[p(\mu + \rho _2)(\mu + \gamma _2)+ \gamma _2 a_2^{*}I_2^{*}]a_3^{*}}{(\mu + \rho _1)(\mu + \gamma _3)a_2^{*}},&\Sigma _{21}&= \frac{a_4^{*}I_2^{*}}{q a_4^{*}I_2^{*} + \mu + \gamma _1}, \end{aligned}$$

$a_i^*=a_i(g(I_2^*))$ is the value of the *i*–th transmission rate ($i=1, \dots , 4$) at the equilibrium $E_2$ (Table 1).

**Table 1:** Summary of the reproduction numbers and their interpretations. The corresponding analytical expressions are given in the indicated equations

Parameter	Symbol	Interpretation	Expression
Basic reproduction number of virus–*i*	$\mathcal {R}_{0i}$	Average number of secondary infections produced by a single individual infected by virus–*i*, in a fully susceptible population.	(12)
Control reproduction number of virus–*i*	$\mathcal {R}_{Ci}$	Average number of secondary infections produced by a single individual infected by virus–*i*, in a fully susceptible population and in the presence of control measures.	(14)
Invasion number of virus–*i* at virus–*j* dominance equilibrium	$\mathcal {R}^{\textrm{inv}}_i$	Average number of secondary infections produced by a single individual infected by virus–*j* in a population where virus–*i* is endemic.	(16), (17)

The following result concerns the local stability of the virus–1 dominance equilibrium:

#### Proposition 4

Let us assume that $\mathcal {R}_{C1} > 1$. If $\mathcal {R}_2^{\text {inv}}<1$, then the virus–1–dominance equilibrium $E_1$ is locally asymptotically stable. If $\mathcal {R}_2^{\text {inv}}>1$, then $E_1$ is unstable.

#### Proof

See Appendix B. $$\square $$

An analogous stability result holds for the virus–2–dominance equilibrium $E_2$.

#### Proposition 5

Let us assume that $\mathcal {R}_{C2} > 1$. If $\mathcal {R}_1^{\text {inv}}<1$, the virus–2–dominance equilibrium $E_2$ is locally asymptotically stable. If $\mathcal {R}_1^{\text {inv}}>1$, then $E_2$ is unstable.

#### Remark 2

The relative magnitudes of the control and invasion numbers depend on whether co–infection is incorporated into the model. In particular:

*(i)*: If there is no co–infection (i.e., $\hat{a}_4=0$), the invasion number $\mathcal {R}_i^{\text {inv}}$ of virus–*i* at the equilibrium $E_j$ assumes the following form:
  18 $$\begin{aligned} \mathcal {R}_i^{\text {inv}} = \frac{a_i(g(I_j^*))}{\mu + \gamma _i} \left( S_j^*+R_j^* \right) = \frac{a_i(g(I_j^*))}{\mu + \gamma _i} \left[ \frac{\mu + \gamma _j}{a_j(g(I_j^*))}+ \frac{\gamma _j}{\mu + \rho _j}I_j^*\right] , \qquad \end{aligned}$$
  with $i,j=1,2, \, i \ne j$. Recalling that the equation (4) implies
  19 $$\begin{aligned} \frac{a_i(g(I_j^*))}{\mu +\gamma _i} = \frac{\pi _i(\hat{c}-c_0)}{\mu +\gamma _i} \left[ 1- \frac{c_1(g(I_j^*))}{\hat{c}-c_0} \right] = \mathcal {R}_{Ci} \left[ 1- \frac{c_1(g(I_j^*))}{\hat{c}-c_0} \right] , \nonumber \\ \end{aligned}$$
  it follows that $\mathcal {R}_i^{\text {inv}}< \mathcal {R}_{Ci}$. Therefore, if co–infection is absent, and virus–*i* cannot establish itself within an entirely susceptible population, it will also be unable to establish itself in a population where virus–*j* is endemic.
*(ii)*: If there is co–infection (i.e, $\hat{a}_4>0$), it may be $\mathcal {R}_{Ci}<1<\mathcal {R}_i^{\text {inv}}$. For example, by setting: $\hat{a}_1=0.3$ $\hbox {d}^{-1}$, $\gamma _1 = 1/5$ $\hbox {d}^{-1}$, $\rho _1= 1/500$ $\hbox {d}^{-1}$, $\hat{a}_2=0.24$ $\hbox {d}^{-1}$, $\gamma _2 = 1/5$ $\hbox {d}^{-1}$, $\rho _1= 1/365$ $\hbox {d}^{-1}$, $\hat{a}_3=0.7$ $\hbox {d}^{-1}$, $\gamma _3 = 1/7$ $\hbox {d}^{-1}$, $\rho _3= 1/365$ $\hbox {d}^{-1}$, $p=0$, $q=1$ and the other parameters as in Table 2 in Section 4, we obtain that $\mathcal {R}_{C1}=1.199$, $\mathcal {R}_{C2}=0.959$, $\mathcal {R}_1^{\text {inv}} = 1.217$ and $\mathcal {R}_2^{\text {inv}} = 1.116$. Therefore, co–infection can facilitate the establishment of virus–*i* in a population where virus–*j* is endemic.

### Forward bifurcation of the disease–free equilibrium

From now on, we are interested in deriving sufficient conditions for the existence and local stability of the co–endemic equilibria

$$\begin{aligned} E_c=(S^{(c)},I_1^{(c)}, I_2^{(c)}, I_3^{(c)}, R_1^{(c)}, R_2^{(c)}, R_3^{(c)},M^{(c)}), \end{aligned}$$

where $I_1^{(c)}$, $I_2^{(c)}$ and $I_3^{(c)}$ are strictly positive. Such equilibria represent steady states in which both diseases coexist in the population.

The following result rules out the existence of co–endemic equilibria when $\mathcal {R}_C< 1$.

#### Theorem 1

Let $\mathcal {R}_{C2}<1$. Then the equilibrium $E_0$ of model (6) undergoes a forward bifurcation at $\mathcal {R}_{C1}=1$. When $\mathcal {R}_{C1}<1$, $E_0$ is locally asymptotically stable whereas when $\mathcal {R}_{C1}>1$, $E_0$ becomes unstable and a locally asymptotically stable endemic equilibrium appears.

#### Proof

We take $\hat{a}_1$ as bifurcation parameter and use Castillo–Chavez and Song bifurcation theorem (Castillo-Chavez and Song 2004, Theorem 4.1) to show that the equilibrium $E_0$ undergoes a forward bifurcation at $\hat{a}_1^{\text {thr}} = \mu + \gamma _1 + a_{1,0}$ (the superscript “thr” indicates the threshold value). The Jacobian matrix $\tilde{J} = J(E_0, \hat{a}_1^{\text {thr}})$ has the following form:

$$\begin{aligned} \tilde{J}= { \begin{bmatrix} -\mu & - (\mu + \gamma _1) & - a_2(0) & -a_3(0) & \rho _1 & \rho _2 & \rho _3 & 0 \\ 0 & 0 & 0 & pa_3(0) & 0 & 0 & 0 & 0 \\ 0 & 0 & a_2(0) - \mu - \gamma _2 & (1-p)a_3(0) & 0 & 0 & 0 & 0 \\ 0& 0 & 0 & -(\mu + \gamma _3) & 0 & 0 & 0 & 0 \\ 0 & \gamma _1 & 0 & 0 & -(\mu + \rho _1) & 0 & 0 & 0\\ 0 & 0 & \gamma _2 & 0 & 0 & -(\mu + \rho _2) & 0 & 0\\ 0 & 0 & 0 & \gamma _3 & 0 & 0 & -(\mu + \rho _3) & 0\\ 0 & k_1/T_a & k_2/T_a & k_3/T_a & 0 & 0 & 0 & -1/T_a\\ \end{bmatrix} } \end{aligned}$$

The right eigenvector *w* of $\tilde{J}$ is

20 $$\begin{aligned} w = \left( -\frac{\mu + \rho _1 + \gamma _1}{\gamma _1}, \frac{\mu + \rho _1}{\gamma _1}, 0, 0, 1, 0, 0, \frac{k_1(\mu + \rho _1)}{\gamma _1} \right) ^\text {t}, \end{aligned}$$

while the left eigenvector *v* is

$$\begin{aligned} v= \left( 0, \frac{\gamma _1}{\mu + \rho _1}, 0, \frac{p\gamma _1(\hat{a}_3-a_{3,0})}{(\mu +\gamma _3)(\mu +\rho _1)}, 0,0,0,0\right) . \end{aligned}$$

Note that the component $w_j$ is nonnegative whenever $(E_0)_j=0$, as required in Castillo-Chavez and Song (2004, Remark 1). The direction of the bifurcation at $\mathcal {R}_{C1}=1$ is determined by the sign of the coefficients $\mathcal {A}$ and $\mathcal {B}$ of the normal form of the transcritical bifurcation (note that in the paper by Castillo-Chavez and Song (2004), such coefficients are denoted by *a* and *b*, respectively). We have:

$$\begin{aligned} \mathcal {A}&:= \sum _{k,i,j = 1}^{8} v_k w_i w_j \frac{\partial ^2 f_k}{\partial x_i \partial x_j}(E_0, \hat{a}_1^{\text {thr}})\\&= -2 \left[ (\hat{a}_2-a_{2,0})\frac{(\mu +\rho _1 + \gamma _1)}{\gamma _1}+k_1(\mu + \rho _1) |a_2'(0)|\right] ,\\ \mathcal {B}&:= \sum _{k,i = 1}^{8} v_k w_i \frac{\partial ^2 f_k}{\partial x_i \partial \hat{a}_1}(E_0, \hat{a}_1^{\text {thr}}) = v_2w_2 \frac{\partial ^2 f_2}{\partial I_1 \partial \hat{a}_1}(E_0, \hat{a}_1^{\text {thr}}) = 1. \end{aligned}$$

Since $\mathcal {A} < 0$ and $\mathcal {B}>0$, a forward bifurcation occurs at $\mathcal {R}_{C1}=1$. $\square$

#### Remark 3

An analogous result to the one in Theorem 1 can be obtained when $\mathcal {R}_{C1}<1$. In this case, the equilibrium $E_0$ undergoes a transcritical forward bifurcation at $\mathcal {R}_{C2}=1$, with the equilibrium $E_0$ unstable and a locally asymptotically stable endemic equilibrium if $\mathcal {R}_{C2}>1$. Then, we can conclude that $E_0$ undergoes a transcritical forward bifurcation at $\mathcal {R}_C=1$.

#### Remark 4

Theorem 1 does not imply that the bifurcating equilibrium is necessarily $E_1$. In fact, if $\hat{a}_4>0$, the bifurcating equilibrium could be the co–endemic equilibrium $E_c$ instead of $E_1$, when the invasion number $\mathcal {R}_2^{\text {inv}}>1$. Consequently, the theorem only rules out the existence of both endemic and co–endemic equilibria when $\mathcal {R}_C<1$.

### Co–endemic equilibria without co–infection

In this subsection, we focus on the case without co–infection (i.e., $\hat{a}_4=0$) and analyse the existence and local stability of a co–endemic equilibrium

$$\begin{aligned} E_c=(S^{(c)},I_1^{(c)}, I_2^{(c)}, R_1^{(c)}, R_2^{(c)},M^{(c)}), \end{aligned}$$

with $I_1^{(c)}, I_2^{(c)}>0$. In this case, the system (6) reduces to the following subsystem:

21a $$\begin{aligned} \dot{S}(t)&= \mu - a_1(M)SI_1 - a_2(M)SI_2 + \rho _1 R_1 + \rho _2R_2 - \mu S,\end{aligned}$$

21b $$\begin{aligned} \dot{I}_1(t)&= a_1(M)SI_1 + a_1(M)R_2I_1 - (\mu + \gamma _1)I_1,\end{aligned}$$

21c $$\begin{aligned} \dot{I}_2(t)&= a_2(M)SI_2 + a_2(M)R_1I_2 - (\mu + \gamma _2)I_2,\end{aligned}$$

21d $$\begin{aligned} \dot{R}_1(t)&= \gamma _1I_1 - a_2(M)R_1I_2 - (\mu + \rho _1)R_1,\end{aligned}$$

21e $$\begin{aligned} \dot{R}_2(t)&= \gamma _2I_2 - a_1(M)R_2I_1 - (\mu + \rho _2)R_2,\end{aligned}$$

21f $$\begin{aligned} \dot{M}(t)&= \frac{1}{T_a}(k_1I_1 + k_2 I_2-M), \end{aligned}$$

By using equations (18) and (19), we get that if $R_{C1}>R_{C2}$, then $\mathcal {R}_1^{\text {inv}}>1$ and the equilibrium $E_2$ is unstable. Hence, if the co–infection rate $\hat{a}_4$ is set equal to zero, the equilibria $E_1$ and $E_2$ cannot be simultaneously locally asymptotically stable.

Concerning the stability of the equilibrium $E_1$, we have that it is locally stable if $\mathcal {R}_2^{\text {inv}}<1$ and destabilises at the bifurcation point $\mathcal {R}_2^{\text {inv}}=1$. In order to prove the existence of a co–endemic equilibrium when $\mathcal {R}_2^{\text {inv}}>1$, we perform a bifurcation analysis on the equilibrium $E_1$, choosing $\hat{a}_2$ as the bifurcation parameter and denoting by $\hat{a}_2^{\text {thr}}$ its value at the bifurcation point where $\mathcal {R}_2^{\text {inv}}=1$. Note that, if $\hat{a}_2 = \hat{a}_2^{\text {thr}}$, the value $a_2^{\text {thr}}(M_1^*)$ at the equilibrium $E_1$ satisfies

22 $$\begin{aligned} a_2^{\text {thr}}(M_1^*) = \frac{a_1^*(\mu + \rho _1)(\mu + \gamma _2)}{(\mu + \gamma _1)(\mu + \rho _1) + \gamma _1a_1^* I_1^*}. \end{aligned}$$

#### Theorem 2

Let $\mathcal {R}_{C1}>\mathcal {R}_{C2}>1$, so that $\mathcal {R}_1^{\text {inv}}>1$. Assume that one of the following two conditions holds:

(i) The virus–2 infectivity rate is such that $\gamma _2 > \tilde{\gamma }_2$, where:
  23 $$\begin{aligned} \tilde{\gamma }_2 := \frac{(\mu +\gamma _1)(\mu + \rho _1)^2}{\gamma _1 (a_1^*- \mu - \gamma _1)(a_1^*)^2} \left[ (a_1^*)^2 + k_2 |a_1'(M_1^*)|(\mu + \gamma _1) \right] - \mu , \end{aligned}$$
(ii) The information coverages $k_1$ and $k_2$ about virus–1 and virus–2 are equal, that is, $k_1=k_2$.

Then, the equilibrium $E_1$ undergoes a transcritical forward bifurcation at $\mathcal {R}_2^{\text {inv}}=1$. When $\mathcal {R}_2^{\text {inv}}<1$, the equilibrium $E_1$ is locally asymptotically stable; when $\mathcal {R}_2^{\text {inv}}>1$, $E_1$ becomes unstable and a locally stable co–endemic equilibrium $E_c$ appears.

#### Proof

We use the bifurcation theorem by Castillo-Chavez and Song (2004) and compute the Jacobian matrix $\tilde{J} = J(E_1)$ at the bifurcation value $\hat{a}_2^{\text {thr}}$:

$$\begin{aligned} \tilde{J} = { \begin{bmatrix} -(\mu + a_1^*I_1^*) & -(\mu +\gamma _1) & -a_2^{\text {thr}}(M_1^*)S_1^* & \rho _1 & \rho _2 & - a_1'(M_1^*)S_1^* I_1^*\\ a_1^*I_1^* & 0 & 0 & 0 & a_1^*I_1^* & a_1'(M_1^*)S_1^* I_1^* \\ 0 & 0 & 0 & 0 & 0 & 0 \\ 0 & \gamma _1 & -a_2^{\text {thr}}(M_1^*)R_1^* & -(\mu + \rho _1) & 0 & 0 \\ 0 & 0 & \gamma _2 & 0 & - (\mu + \rho _2 + a_1^*I_1^*) & 0 \\ 0 & \frac{k_1}{T_a} & \frac{k_2}{T_a} & 0 & 0 & -\frac{1}{T_a} \end{bmatrix}, } \end{aligned}$$

where $a_1'(M_1^*) = \frac{\text {d}a_1}{\text {d}M}\Big |_{M_1^*}$ is negative, since $a_1$ is monotonically decreasing in *M*.

The matrix $J(E_1)$ has one simple zero eigenvalue and all the other eigenvalues with negative real parts, so the hypothesis *A*1 of Castillo-Chavez and Song (2004, Theorem 4.1) is satisfied. The left eigenvector *v* corresponding to the zero eigenvalue is

$$\begin{aligned} v = (0, 0, 1, 0, 0, 0). \end{aligned}$$

Furthermore, since the right eigenvector $w=(w_1, w_2, w_3, w_4, w_5, w_6)$ has to satisfy the condition $v \cdot w = 1$, we get $w_3 =1$. The other components of *w* have to be computed from the following linear system:

24a $$\begin{aligned}&-(\mu + a_1^*I_1^*)w_1 - (\mu + \gamma _1)w_2 + \rho _1 w_4 + \rho _2w_5 \nonumber \\&\quad + |a_1'(M_1^*)|S_1^* I_1^*w_6 = a_2^{\text {thr}}(M_1^*)S_1^* \end{aligned}$$

24b $$\begin{aligned}&a_1^*I_1^* (w_1 + w_5) + |a_1'(M_1^*)|S_1^* I_1^*w_6 =0 \end{aligned}$$

24c $$\begin{aligned}&\gamma _1w_2 - (\mu + \rho _1)w_4 = a_2^{\text {thr}}(M_1^*)R_1^*\end{aligned}$$

24d $$\begin{aligned}&(\mu + \rho _2 + a_1^*I_1^*)w_5 = \gamma _2\end{aligned}$$

24e $$\begin{aligned}&k_1w_2 - w_6 = - k_2 \end{aligned}$$

From equations (24d), (24e), (24e), we derive:

25 $$\begin{aligned} w_4 = - \frac{\gamma _1a_2^{\text {thr}}(M_1^*)I_1^*}{(\mu + \rho _1)^2} + \frac{\gamma _1}{\mu + \rho _1}w_2, \quad w_5= \frac{\gamma _2}{\mu + \rho _2 + a_1^*I_1^*}, \quad w_6 = k_2 + k_1w_2, \nonumber \\ \end{aligned}$$

and using equations (24b) and (25), we can compute $w_1$ depending on $w_2$:

26 $$\begin{aligned} w_1 = \left[ \frac{k_1|a_1'(M_1^*)|(\mu +\gamma _1)}{(a_1^*)^2}\right] w_2 + \left[ \frac{|a_1'(M_1^*)|(\mu +\gamma _1)k_2}{(a_1^*)^2}- \frac{\gamma _2}{\mu + \rho _2 + a_1^*I_1^*}\right] . \nonumber \\ \end{aligned}$$

Furthermore, by adding equations (24a), (24b), (24c), (24d), we get:

27 $$\begin{aligned} -\mu (w_1 + w_2 + w_4 + w_5)= a_2^{\text {thr}}(M_1^*)(S_1^* + R_1^*) - \gamma _2. \end{aligned}$$

Remembering that $a_2^{\text {thr}}$ satisfies $\mathcal {R}_2^{\text {inv}}=1$, we can get the following condition:

$$\begin{aligned} \mathcal {R}_2^{\text {inv}}= \frac{a_2^{\text {thr}}(S_1^* + R_1^*)}{\mu + \gamma _2}=1 \quad \implies \quad a_2^{\text {thr}}(S_1^* + R_1^*) - \gamma _2 = \mu , \end{aligned}$$

that can be substituted in (27) to get the following equation to find $w_2$:

28 $$\begin{aligned} w_1 + w_2 + w_4 + w_5 = -1. \end{aligned}$$

Note that the components $w_3$ and $w_5$ of the eigenvector *w* are nonnegative, as required by the hypothesis *A*2 of Castillo-Chavez and Song (2004, Theorem 4.1). By using (26), (25) and (28), we can compute $w_2$:

29 $$\begin{aligned} w_2 = \frac{\gamma _1a_2^{\text {thr}}(M_1^*)(a_1^*)^2I_1^*- k_2|a_1'(M_1^*)|(\mu + \gamma _1)(\mu + \rho _1)^2-(\mu +\rho _1)^2(a_1^*)^2}{(\mu +\rho _1)^2(a_1^*)^2+k_1|a_1'(M_1^*)|(\mu +\gamma _1)(\mu +\rho _1)^2+\gamma _1(\mu +\rho _1)(a_1^*)^2}. \nonumber \\ \end{aligned}$$

The direction of the bifurcation at the value $\mathcal {R}_2^{\text {inv}}=1$ is determined by the sign of the coefficients $\mathcal {A}$ and $\mathcal {B}$. We have:

$$\begin{aligned} \mathcal {B} := \sum _{k,i = 1}^{6} v_k w_i \frac{\partial ^2 f_k}{\partial x_i \partial \hat{a}_2}(E_1, a_2^{\text {thr}}) = \partial _{\hat{a}_2} a_2(M_1^*)(S_1^*+R_1^*) > 0, \end{aligned}$$

and:

$$\begin{aligned} \mathcal {A}&:= \sum _{k,i,j = 1}^{6} v_k w_i w_j \frac{\partial ^2 f_k}{\partial x_i \partial x_j}(E_1, a_2^{\text {thr}}) =a_2^{\text {thr}}(M_1^*)(w_1 + w_4)- |a_1'(M_1^*)|(S_1^* + R_1^*)w_6 \\&= -a_2^{\text {thr}}(M_1^*)\left( 1+w_2 + \frac{\gamma _2}{\mu + \rho _2 + a_1^*I_1^*} \right) - |a_1'(M_1^*)|(S_1^* + R_1^*)(k_2+k_1w_2) \\&= -\mathcal {C}_1 w_2 -\mathcal {C}_0, \end{aligned}$$

where

$$\begin{aligned} {\mathcal {C}_1}&:= a_2^{\text {thr}}(M_1^*) + k_1|a_1'(M_1^*)|(S_1^*+R_1^*),\\ {\mathcal {C}_0}&:= a_2^{\text {thr}}(M_1^*)+ \frac{a_2^{\text {thr}}(M_1^*)\gamma _2}{\mu + \rho _2 + a_1^*I_1^*}+ k_2|a_1'(M_1^*)|(S_1^*+R_1^*). \end{aligned}$$

Note that both $\mathcal {C}_1$ and $\mathcal {C}_0$ are positive. If condition *(i)* is satisfied, then $\gamma _2 > \tilde{\gamma }_2$ and $w_2>0$. This implies that $\mathcal {A}<0$ and the direction of the bifurcation is forward. If condition *(ii)* holds, it is easy to see from equation (29) that $w_2>-1$. Thus, we obtain:

$$\begin{aligned} \mathcal {A}< {\mathcal {C}_1 - \mathcal {C}_0} = - \frac{a_2^{\text {thr}}(M_1^*)\gamma _2}{\mu + \rho _2 + a_1^*I_1^*}<0, \end{aligned}$$

and the bifurcation is forward. $\square$

#### Remark 5

*(i)*: The analogous result can be stated for the equilibrium $E_2$ by assuming $\mathcal {R}_{C2}> \mathcal {R}_{C1} > 1$, in order to have $\mathcal {R}_2^{\text {inv}} > 1$. In this case, by taking $\hat{a}_1$ as bifurcation parameter, we can give sufficient conditions for the occurrence of a forward bifurcation at $\mathcal {R}_1^{\text {inv}} = 1$, with the equilibrium $E_2$ that is locally asymptotically stable when $\mathcal {R}_1^{\text {inv}} < 1$ and unstable when $\mathcal {R}_1^{\text {inv}} > 1$, with the bifurcating co–endemic equilibrium $E_c$ being locally asymptotically stable.
*(ii)*: Theorem 2 gives sufficient conditions guaranteeing that $\mathcal {A} < 0$. However, extensive numerical simulations (not shown here) suggest that for the set of epidemiological parameters considered in this study, the coefficient $\mathcal {A}$ is negative for any values of the information–related parameters.

## Effects of information–induced behavioural changes

### Parameter values and initial conditions

**Fig. 3:**
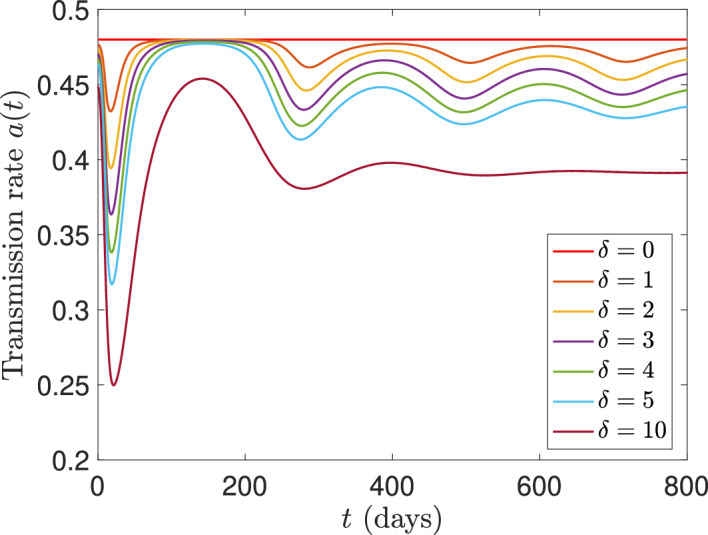
Time profiles of the transmission rate $a(t):= a_i(t) = (\hat{a}_{i}- a_{i,0})/(1+\delta M(t))$, for the population reactivity rate $\delta$ varying between 0 to 10 and baseline transmission rate $\hat{a}_i = 0.6$ ($i =1,2,3$)

A complete data set from field cases is not yet available for model (6). Therefore, the epidemiological parameter values are taken from Fahlena et al. (2022). In particular, as was done in Fahlena et al. (2022), we choose $\hat{a}_1 = \hat{a}_2 = \hat{a}_3$ and denote them as $\hat{a}$. The value of the average information delay $T_a$ is borrowed from Buonomo and Della Marca (2020), whose estimate relies on the first COVID–19 wave in Italy. The information–independent reduction rates $a_{1,0}$, $a_{2,0}$, $a_{3,0}$ and $a_{4,0}$ are guessed to be $20\%$ of the corresponding non–behavioural parameters.

Regarding the information coverages $k_1$, $k_2$ and $k_3$, we consider two different sets of values. The first set is given by $k_1 = k_2 = k_3 = 0$ and describes the situation in which the population does not reduce its social contacts, either because it does not perceive the two diseases as a health risk or because it is not informed about their prevalence. We denote this scenario as the *non–behavioural* scenario.

The second set of values aims to represent the *behavioural* scenario in which the population modifies its contact patterns due to information. We choose $k_1 = k_2 = 0.8$ as in Buonomo and Della Marca (2020), while we assign a lower value to the co–infection information coverage, say $k_3 = 0.2$, since usually the official data do not provide information about the number of co–infected (World Health Organisation 2024a, b) and the perception of co–infected cases is mostly based on rumours.

Concerning the value of the population reactivity rate $\delta$, for $\delta$ varying in [0, 10], the transmission rate *a*(*M*(*t*)) ranges between 0.45 and 0.6 after an initial transient (see Figure 3). Therefore, taking $\delta = 1$ allows to observe variations in the transmission rate *a*(*M*(*t*)) and, at the same time, to avoid that the transmission rate becomes negligible.

Finally, we assume that only a small number of infected is initially present in the community, say $I_1(t_0) = 0.005$ and $I_2(t_0) = 0.003$, and that at the initial time $t_0$, the individuals base their behaviour only on current data, so that

30 $$\begin{aligned} M(t_0) = k_1I_1(t_0) + k_2I_2(t_0)+ k_3I_3(t_0). \end{aligned}$$

All the parameter values of the model, together with the initial conditions, are reported in Table 2.

**Table 2:** Parameter definitions of model (6), together with the values and sources used to simulate the behavioural scenario in Figure 4

Quantity	Description	Value	Source
$\mu$	Recruitment and natural death rate	$3.9 \cdot 10^{-5}~\text {d}^{-1}$	(Fahlena et al. 2022)
$\hat{a}_i$	Baseline transmission rate of virus–*i* ($i = 1,2$)	Varies	—
$\hat{a}_3$	Baseline transmission rate of co–infected individuals	Varies	—
$\hat{a}_4$	Baseline co–infection rate	$5~\text {d}^{-1}$	(Fahlena et al. 2022)
$a_{i,0}$	Info-independent transmission rate of virus–*i* ($i = 1,2$)	$0.2\, \hat{a}_i$	Assumed
$a_{3,0}$	Info-independent transmission rate of co–infected individuals	$0.2\, \hat{a}_3$	Assumed
$a_{4,0}$	Info-independent co–infection rate	$0.2\, \hat{a}_4$	Assumed
*p*	Probability that a co–infected individual transmits virus–1 to a susceptible	0.20	Assumed
*q*	Probability that an individual in $I_1$ gets co–infected after a contact with $I_2$	0.20	Assumed
$\gamma _i$	Recovery rate from virus–*i*	$0.14~\text {d}^{-1}$	(Fahlena et al. 2022)
$\gamma _3$	Recovery rate from co–infection	$0.14~\text {d}^{-1}$	(Fahlena et al. 2022)
$\rho _i$	Loss of immunity rate towards virus–*i*	$0.0027~\text {d}^{-1}$	(Fahlena et al. 2022)
$\rho _3$	Loss of immunity rate towards co–infection	$0.0027~\text {d}^{-1}$	(Fahlena et al. 2022)
$k_i$	Information coverage about virus–*i*	0.80	(Buonomo and Della Marca 2020)
$k_3$	Information coverage about co–infection	0.2	Assumed
$\delta$	Population reactivity rate	1	Assumed
$T_a$	Average information delay	3 days	(Buonomo and Della Marca 2020)

**Fig. 4:**
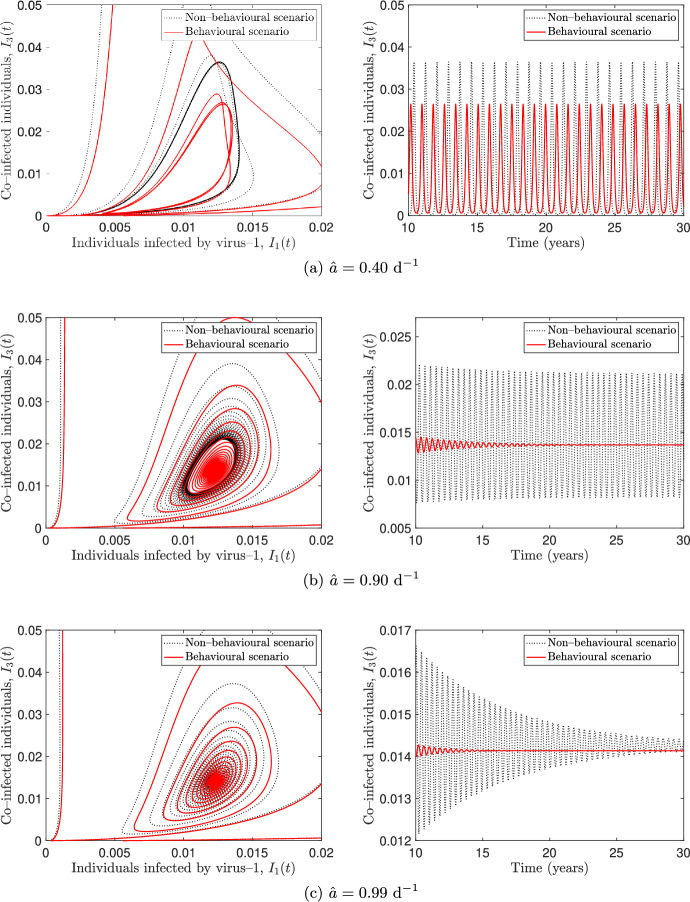
Numerical simulations for the system (6) with different values of $\hat{a}$ and the other parameters as in Table 2. The behavioural scenario ($k_1 = k_2 =0.8$, $k_3 = 0.2$, solid lines) is compared with the non–behavioural scenario ($k_1 = k_2 = k_3 = 0$, dotted lines). The panels on the left show the projection of the solution curve in the $I_1I_3$ plane and the panels on the right show the co–infected individuals $I_3(t)$ depending on time. The time intervals in the right panels are chosen for illustrative purposes

### Effects of information–induced behavioural changes on the oscillatory dynamics

We numerically explore the solutions of model (6) for different values of the baseline transmission rate $\hat{a}$ and compare them in the behavioural and non–behavioural scenarios introduced in the last section. The simulations, performed by using the MATLAB solver ode45, are shown in Figure 4, where the solutions in the behavioural scenario (solid lines) are overlapped to those obtained in the non–behavioural one (dotted lines).

In both scenarios, a given value $\hat{a}^*$ looks like a bifurcation point that marks the transition between sustained oscillations (Figure 4a) and damped oscillations towards a steady state (Figure 4c) in which the co–infection is endemic. The simulations in Figure 4 also show that the threshold value $\hat{a}^*$ is lower in the behavioural scenario than in the non–behavioural one. Therefore, for some values of $\hat{a}$ the non–behavioural scenario can exhibit sustained oscillations while the solutions of the behavioural one converge to a co–endemic equilibrium (see Figure 4b). In view of this, we can conclude that information–induced reduction of social contacts alone may produce a stabilizing effect.

**Fig. 5:**
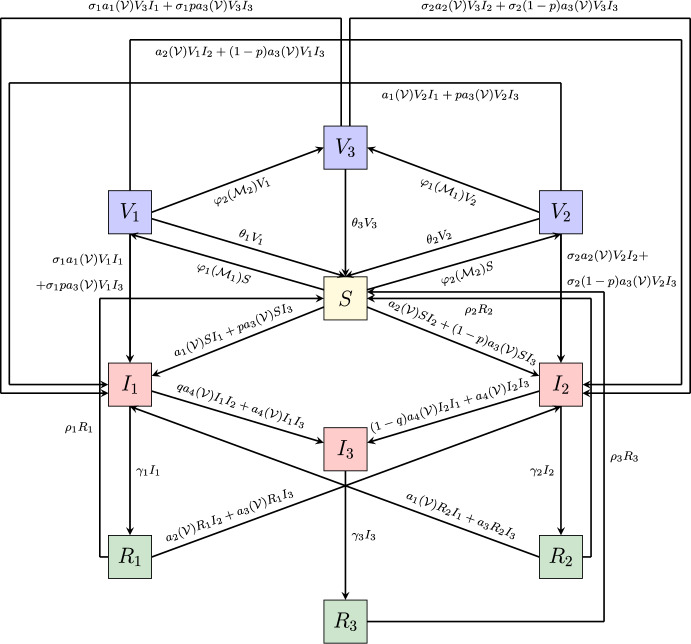
Flowchart of model (6), extended to include vaccination. The system of balance equations for the extended model is provided in (48) (Appendix C). Recruitment into the susceptible compartment and natural death in each compartment are not shown in the diagram

## Extension to vaccine–preventable respiratory viruses

### Modelling vaccination and its impact on social distancing compliance

So far, we have considered the case where only non–pharmaceutical strategies were put in place to contain the epidemic. However, for many respiratory viruses, vaccines are currently available. Human behaviour may play a major role in the success of vaccination campaigns. It is recognised that vaccination behaviour may be affected by information and rumours gathered on perceived risks of getting the disease and the perceived risks of being affected by vaccine side effects (Wang et al. 2016). For example, individuals may be more prone to vaccinate when the prevalence of a virus is high and less prone to vaccinate when the prevalence decreases, as the fear of vaccine side effects begins to prevail in the benefit–cost balance (Suryawanshi and Biswas 2023; Kaushal et al. 2023). Furthermore, the implementation of vaccination policies may be combined with non–pharmaceutical containment measures (such as social distancing and wearing facial masks) as occurred during the COVID–19 pandemic. However, when these two control methods coexist, adherence to social distancing may be negatively influenced by high vaccination coverage. In fact, knowing that a high proportion of the population is vaccinated can encourage individuals to be less careful about their behaviour and to relax social distancing, relying on the efficacy of vaccines. As a consequence, such relaxation may increase viral infections (Andersson et al. 2021; Buonomo et al. 2022).

In the literature, some co–infections models that include vaccination can be found (Majeed et al. 2023; Ojo et al. 2022). However, the effects of human behavioural changes are not considered. For this reason, we extend model (6) by including vaccination. Precisely, we assume that two vaccines, say vax–1 and vax–2, are available for the disease caused by virus–1 and virus–2, respectively. Such vaccines are assumed to be no combo–like, in the sense that vax–*i* has no effect on the propagation of virus–*j* and vice-versa. A practical example is provided by the influenza and SARS-CoV-2 vaccines, which do not offer cross-protective effects (Liang et al. 2024; Del Riccio et al. 2024). The extended model is used to study two different phenomena produced by human behavioural changes: the prevalence–induced vaccination and the vaccination–induced relaxation of social distancing. Specifically, as in Buonomo et al. (2022), we assume that: (i) the choice to vaccinate against each virus is partially voluntary and depends on information about its prevalence; (ii) the choice of relaxing social distancing depends on the information about the level of vaccination compliance.

The extended model is obtained by adding three new compartments to model (6): $V_1$, $V_2$ and $V_3$, representing the individuals vaccinated only against virus–1, only against virus–2 and against both viruses, respectively. The total population, denoted by *N*(*t*), is now divided into ten disjointed compartments and

$$\begin{aligned} N(t) = S(t)+ \sum _{i = 1}^3 I_i(t) + \sum _{i = 1}^3 R_i(t) + \sum _{i = 1}^3 V_i(t). \end{aligned}$$

For the sake of brevity, the balance equations of the extended model are given in (48) of Appendix C, while its flow chart is shown in Figure 5.

The current and past values of virus–1 and virus–2 epidemics, together with current and past vaccination data, are contained in the following three information indexes:

31 $$\begin{aligned} \mathcal {M}_1(t)&= \int _{0}^{+\infty } g_1(I_1(t- \tau ), I_3(t- \tau ))\text {Erl}_{1,1/T_1}(\tau )d\tau , \end{aligned}$$

32 $$\begin{aligned} \mathcal {M}_2(t)&= \int _{0}^{+\infty } g_2(I_2(t - \tau ), I_3(t - \tau ))\text {Erl}_{1,1/T_2}(\tau )d\tau , \end{aligned}$$

33 $$\begin{aligned} \mathcal {V}(t)&= \int _{0}^{+\infty } h(V_1(t - \tau ), V_2(t - \tau ), V_3(t - \tau ))\text {Erl}_{1,1/T_v}(\tau )d\tau , \end{aligned}$$

where $g_1$ (resp. $g_2$) is a message function that describes how knowledge of quantities $I_1$ and $I_3$ (resp. $I_2$ and $I_3$) influences the individual choice to get vaccinated against virus–1 (resp. virus–2). Analogously, the function *h* relates the knowledge of quantities $V_1$, $V_2$ and $V_3$ to the individual’s choice of relaxing social distance. The functions “$\text {Erl}_1$” denote first–order Erlang distribution memory kernels and parameters $T_1$, $T_2$ and $T_v$ may be interpreted as the average time delays of the collected information about the prevalence of virus–1, virus–2 and the level of vaccination compliance, respectively.

Analogously with equation (3), we consider the following message functions:

34 $$\begin{aligned} g_1(I_1, I_3)&= k_1 I_1 + k_3I_3,\end{aligned}$$

35 $$\begin{aligned} g_2(I_2, I_3)&= k_2 I_2 + k_3I_3, \end{aligned}$$

36 $$\begin{aligned} h(V_1, V_2, V_3)&= \tilde{k}_1V_1 + \tilde{k}_2V_2 +\tilde{k}_3V_3, \end{aligned}$$

where $k_1$, $k_2$ and $k_3$ are, respectively, the information coverage about the prevalence of virus–1, virus–2 and co–infection and $\tilde{k}_1$, $\tilde{k}_2$ and $\tilde{k}_3$ are the information coverages about the vaccination data.

We assume that individuals are immunised against virus–*i* at a vaccination rate $\varphi _i$ and that is dependent on the information index $\mathcal {M}_i(t)$, given in (31) and (32). Moreover, the transmission rates $a_j$, $j = 1,2,3$, and the co–infection rate $a_4$ are assumed to depend on the information index $\mathcal {V}(t)$, given in (33). The specific functional forms adopted to describe such information–dependent rates are discussed below.

*i) Vaccination rates.* The rate $\varphi _i$ is given by:

37 $$\begin{aligned} \varphi _i(\mathcal {M}_i) = \varphi _{0,i} + \varphi _{1,i}(\mathcal {M}_i), \qquad i =1,2. \end{aligned}$$

The first term on the r. h. s., $\varphi _{0,i}$, is a positive constant that represents the percentage of susceptibles who choose to get vaccinated independently of the information (for example, people strongly in favour of vaccines, health workers, fragile individuals for whom health authorities strongly recommend vaccination, etc.). The second term on the r.h.s. of (37), $\varphi _{1,i} (\mathcal {M}_i)$, represents the fraction of susceptibles whose vaccination choice is influenced by the information. In analogy with the function $c_1(M)$ in (4), we require that $\varphi _{1,i}$ is a non–negative, continuous, differentiable and increasing function, $\varphi _{1,i}(0) = 0$ and $\text {sup}(\varphi _{1,i}(\mathcal {M}_i)) < \varphi _{\text {max}} - \varphi _{0,i}$, where $\varphi _{\text {max}}$ is a positive upper bound. These requirements are satisfied by the following Holling type II functional:

$$\begin{aligned} \varphi _{1,i}(\mathcal {M}_i) = (\varphi _{\text {max}}- \varphi _{0,i}) \frac{D_i \mathcal {M}_i}{1+ D_i \mathcal {M}_i} \qquad \text {for } i =1,2, \end{aligned}$$

where $D_i$ is a positive constant that shapes individual reactivity to voluntary vaccination against virus–*i* (Buonomo et al. 2008).

*ii) Transmission and co–infection rates.* Motivated by the discussion in subsection 5.1 and following the notation adopted in Section 2, we set:

38 $$\begin{aligned} a_j(\mathcal {V}) = \pi _{j} \big [ \hat{c} - c_0 + c_1(\mathcal {V}) \big ]. \end{aligned}$$

We require that the function $c_1$ in (38) is non–negative, continuous, differentiable and increasing, $c_1(0) = 0$ and $\text {sup}(c_1(\mathcal {V})) \le c_0 < \hat{c}$. These requirements are satisfied by the following Holling type II functional:

39 $$\begin{aligned} c_1(\mathcal {V}) = c_0 \frac{\tilde{D}\mathcal {V}}{1+\tilde{D}\mathcal {V}}, \end{aligned}$$

where $\tilde{D}$ is a positive constant tuning of the reactivity of individuals to relax their social distancing and other non–pharmaceutical interventions.

Note that the difference between formulation (4) and (38) is that in the former, the rate $a_j$ decreases with the index *M*, while in the latter the rate $a_j$ increases with the index $\mathcal {V}$, since it causes the increase in the contact rate due to the relaxation of non–pharmaceutical measures.

Further details on the extended model, including the computation of the control reproduction number $\mathcal {P}_c$ and the local stability of the disease–free equilibrium $E^*$ can be found in Appendix D.

### Parameter values and initial conditions

**Table 3:** Definitions of the vaccination-related parameters contained in the extended model (48) and the values used to simulate the relaxation scenario. Except for the value of $\tilde{k}_3$, such values have been used in previous theoretical investigations (Buonomo et al. 2022; Buonomo and Della Marca 2019) and are mainly guessed

Quantity	Description	Value
$\tilde{k}_i$	Information coverage about vax–*i* ($i = 1,2$)	0.95
$\tilde{k}_3$	Information coverage about individuals that received both vaccines	$0.5 \cdot \tilde{k}_1$
$\sigma _i$	Factor of ineffectiveness of vax–*i*	0.15
$\theta _i$	Rate of loss of immunity given by vax–*i* ($i = 1,2$)	$0.0027~\text {d}^{-1}$
$\theta _3$	Rate of loss of immunity given by both vaccines	$0.0027~\text {d}^{-1}$
$\varphi _{0,i}$	Fraction of susceptibles choosing to receive vax–*i* regardless of information	0.004
$\varphi _{\text {max}}$	Upper bound of the vaccination rates	0.99
$D_i$	Population reactivity to voluntarily receive vax–*i* ($i = 1,2$)	2.5
$\tilde{D}$	Population reactivity to relax social distancing	0.25
$T_i$	Average information delay about virus–*i* prevalence	35 days
$T_v$	Average information delay about vaccination data	60 days

We take the values in Table 2 for all the parameters that the extended model (48) shares with model (6). The values of the vaccination–related parameters, except for $\tilde{k}_3$, are taken from previous theoretical investigations (Buonomo et al. 2022; Buonomo and Della Marca 2019) and are mainly guessed. Regarding the information coverages $\tilde{k}_1$, $\tilde{k}_2$ and $\tilde{k}_3$ we consider two different sets of values, as done in subsection 4.1. The first set is given by $\tilde{k}_1 = \tilde{k}_2 = \tilde{k}_3 = 0$ and describes the *no–relaxation* scenario in which the relaxation of social distancing is neglected. The second set of values represents the *relaxation scenario* and is given by $\tilde{k}_1 = \tilde{k}_2 = 0.95$ and $\tilde{k}_3 = 0.5\,\tilde{k}_1$. The reduced value of the information coverage $\tilde{k}_3$ reflects the circumstance that usually the population is not fully aware of the number of vaccinated individuals against both viruses, because such a number may be not provided by health agencies. For example, data on co-vaccinated individuals was not provided for the recent vaccination campaign against COVID–19 in the US (Centers For Disease Control and Prevention 2024b, a). Nonetheless, $\tilde{k}_3$ is not zero, since the population awareness depends not only on official information but also on rumours.

As for the initial conditions, reasoning as in subsection 4.1, we set:

40 $$\begin{aligned} \mathcal {M}_1(t_0)&= k_1I_1(t_0)+ k_3I_3(t_0), \end{aligned}$$

41 $$\begin{aligned} \mathcal {M}_2(t_0)&= k_2I_2(t_0)+ k_3I_3(t_0),\end{aligned}$$

42 $$\begin{aligned} \mathcal {V}(t_0)&= \tilde{k}_1V_1(t_0)+ \tilde{k}_2V_2(t_0) + \tilde{k}_3V_3(t_0). \end{aligned}$$

The guessed initial values are $I_1(t_0) = 0.005$, $I_2(t_0) = 0.003$, $V_1(t_0) = V_2(t_0) = 0.1$.

All the vaccination–related parameters contained in the extended model, together with the values used to simulate the relaxation scenario, are reported in Table 3.

### Effects of vaccination–induced relaxation of social distancing on oscillatory dynamics

**Fig. 6:**
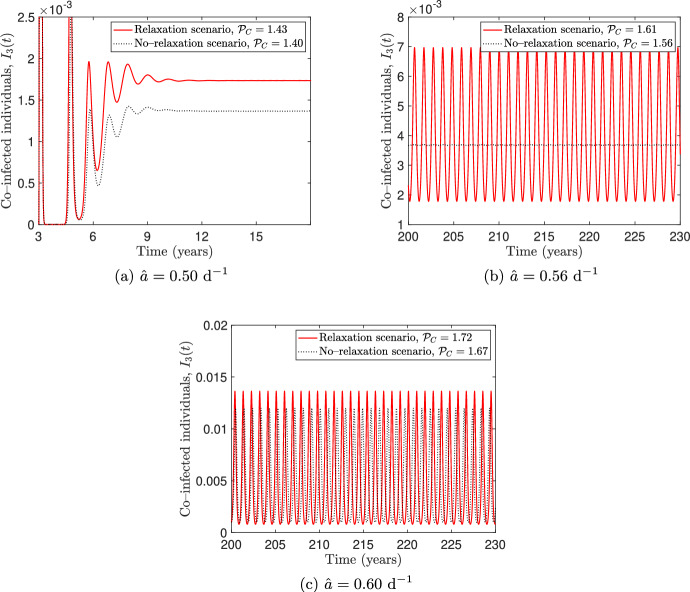
Numerical simulations for the extended model (48) for different values of $\hat{a}$. The other parameter values are taken as in Tables 2 and 3. The relaxation scenario ($\tilde{k} = 0.95$ and $\tilde{k}_3 = 0.5 \, \tilde{k}$, solid lines) is compared with the no–relaxation scenario ($\tilde{k} = \tilde{k}_3 = 0$, dotted lines). The time intervals are chosen for illustrative purposes

As already done in subsection 4.2, we show the solutions of the extended model for different values of the baseline transmission rate $\hat{a}$ and compare the relaxation scenario with the no–relaxation one. Figure 6 shows the comparison between the solutions obtained in the relaxation scenario (solid lines) and in the no–relaxation scenario (dotted lines), with the corresponding control reproduction numbers $\mathcal {P}_c$, computed using equation (50) in Appendix D. From Figure 6, we observe that the extended model admits a threshold value, say $\mathcal {P}_c^*$, such that when $\mathcal {P}_c$ crosses $\mathcal {P}_c^*$, the solutions pass from converging towards a co–endemic steady state (panel 6a) to exhibiting sustained oscillations over time (panel 6c).

However, it follows trivially from the equation (50) that the value of $\mathcal {P}_c$ in the no–relaxation scenario is strictly smaller than in the relaxation scenario. Consequently, for some values of $\hat{a}$ we have $\mathcal {P}_c < \mathcal {P}_c^*$ in the no–relaxation scenario, while in the relaxation scenario we have $\mathcal {P}_c > \mathcal {P}_c^*$. This implies that the two scenarios have different long–time dynamics: sustained oscillations occur in the relaxation scenario, while damped oscillations occur in the no–relaxation one (panel 6b). This suggests that the vaccination–induced relaxation of social distancing may destabilise the model dynamics.

### Impact of vaccination–related information parameters on disease prevalence, incidence and vaccinations

We investigate the impact of both the average information delay, $T_v$, and the information coverage, $\tilde{k}$ (where $\tilde{k}:= \tilde{k}_1 = \tilde{k}_2$), on disease prevalence, incidence and vaccinations. To this aim, we consider the following relevant quantities: *i)* the *maximum total prevalence* MP(*t*), i.e., the highest value of total prevalence in the time frame $[t_0, t]$; *ii)* the *total cumulative incidence* CI(*t*), i.e., the cumulative number of new cases of virus–1 and virus–2 in the time interval $[t_0,t]$; and *iii)* the *cumulative vaccinations* CV(*t*), i.e., the cumulative number of vax–1 and vax–2 vaccines administered in the time interval $[t_0, t]$. They can be expressed as follows:

$$\begin{aligned} \text {MP}(t)&= \text {max}(I_1(\tau )+ I_2(\tau )+I_3(\tau )), \quad \tau \in [t_0,t],\\ \text {CI}(t)&= \int _{t_0}^t \big \{ a_1(\mathcal {V}(\tau ))I_1(\tau ) \big [ S(\tau ) + R_2(\tau ) + \sigma _1 V_1(\tau ) + V_2(\tau ) + \sigma _1 V_3(\tau ) \big ] \\&\quad + a_2(\mathcal {V}(\tau ))I_2(\tau ) \big [ S(\tau ) + R_1(\tau ) + V_1(\tau ) + \sigma _2 V_2(\tau ) + \sigma _3 V_3(\tau ) \big ] \\&\quad + a_3(\mathcal {V}(\tau ))I_3(\tau ) \big [ S(\tau ) + R_1(\tau ) + R_2(\tau ) + p \big (\sigma _1 V_1(\tau ) + V_2(\tau ) \\&\quad + \sigma _1 V_3(\tau ) \big ) + (1-p) \big ( V_1(\tau ) + \sigma _2 V_2(\tau ) + \sigma _2 V_3(\tau ) \big ) \big ] \\&\quad + a_4(\mathcal {V}(\tau )) \big [ I_1(\tau ) I_2(\tau ) + I_1(\tau ) I_3(\tau ) + I_2(\tau ) I_3(\tau ) \big ] \big \} \, d\tau ,\\ \text {CV}(t)&= \int _{t_0}^t \big \{ \varphi _1(\mathcal {M}_1(\tau ))\big [ S(\tau ) + V_2(\tau ) \big ] + \varphi _2(\mathcal {M}_2(\tau )) \big [ S(\tau ) + V_1(\tau ) \big ] \big \} \, d\tau . \end{aligned}$$

**Fig. 7:**
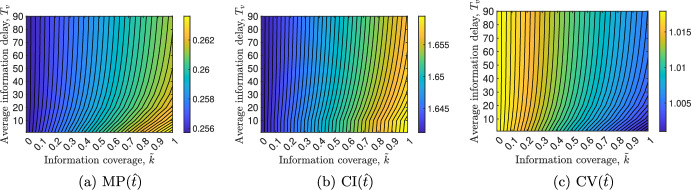
Contour plots of CI$(\hat{t})$, CV$(\hat{t})$ and MP$(\hat{t})$, evaluated at $\hat{t} = 120$ days, versus the information coverage $\tilde{k}$ (where $\tilde{k}:= \tilde{k}_1 = \tilde{k}_2$) and the average information delay $T_v$. The values of the parameters are reported in Tables 2 and 3 except for the baseline transmission rate, which is set $\hat{a} = 0.55 \text { d}^{-1}$

For illustrative purposes, we fix $\hat{t} = 120$ days and perform the contour plots of CI$(\hat{t})$, CV$(\hat{t})$ and MP$(\hat{t})$ as the parameters $\tilde{k}$ and $T_v$ vary. Figure 7 shows that all the above mentioned quantities depend mainly on the information coverage $\tilde{k}$, as it can be deduced by the almost vertical contour lines. Conversely, all of them become more sensitive to variations in $T_v$ if $\tilde{k} > 0.7$. Figure 7 also shows that CI$(\hat{t})$ and MP$(\hat{t})$ monotonically increase with $\tilde{k}$, and both of them reach their respective maximum values when $\tilde{k} =1$. This result is consistent with the fact that the transmission rates $a_1$, $a_2$ and $a_3$ as well as the co–infection rate *b* increase monotonically with the information index $\mathcal {V}$, and therefore they increase monotonically with $\tilde{k} = \tilde{k}_1 = \tilde{k}_2$ in virtue of (33) and equation (36).

As for the cumulative vaccinations CV$(\hat{t})$, we find a first glance contradictory result since CV$(\hat{t})$ decreases with $\tilde{k}$ (panel 7c) although the vaccination rates $\varphi _1$ and $\varphi _2$ increase monotonically with the number of infected, which in turn increase with $\tilde{k}$. However, observe that CV$(\hat{t})$ is the result of two contrasting phenomena. From on one hand, the number of vaccinated increases as the prevalence of the two viruses increases; on the other hand, CV$(\hat{t})$ decreases due to both the waning effect of immunisation and the high number of infected, for which vaccination is useless. The fact that the quantity CV$(\hat{t})$ decreases with $\tilde{k}$ until its minimum value for $\tilde{k}=1$ suggests that the second phenomenon is the prevailing one.

We finally remark that, although simultaneous vaccination against different respiratory viruses is possible (Centers For Disease Control and Prevention 2024c), here we do not consider the possibility of simultaneous administration of the two vaccines. However, many numerical simulations – not reported here – show that the inclusion of simultaneous vaccination does not produce any relevant qualitative difference when compared to the solutions of the extended model.

**Fig. 8:**
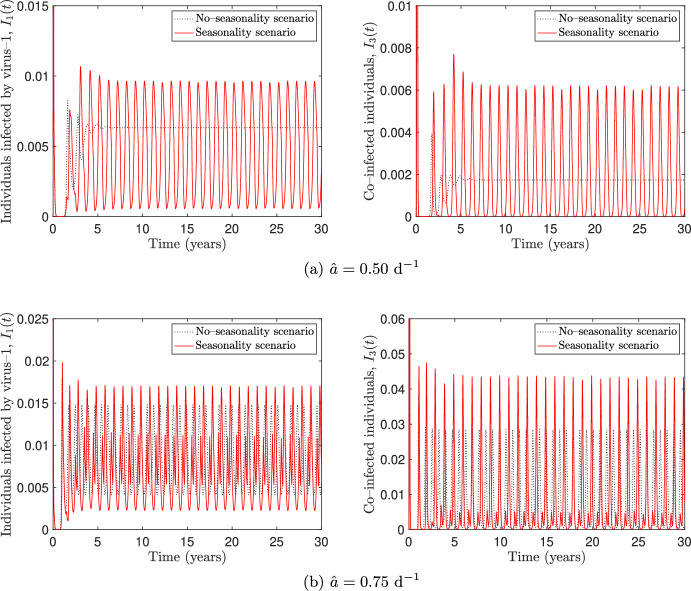
Numerical simulations in the seasonality (solid red lines) and no–seasonality (dotted black lines) scenarios, for different values of the baseline transmission rate $\hat{a}$ and for $\epsilon _l = 0.25$ ($l = 1, \dots , 6$). The values of the other parameters are reported in Tables 2 and 3

### Effects of seasonality on the spread of co–infections

Respiratory viruses can be affected by seasonal variations due to both environmental and human behavioural factors. On the one hand, low temperatures, combined with very high or very low humidity levels, may enhance the viability and transmissibility of virus droplets in the environment and negatively affect the host’s airway antiviral defences (Moriyama et al. 2020). On the other hand, the lower temperatures during the winter season in temperate climates increase indoor person-to-person contacts which could lead to enhanced virus transmission (Schweizer et al. 2007). Furthermore, travel and social gatherings during holidays favour person–to–person contacts and thus the contagion outside the usual circle of contacts. Therefore, human behaviour may play a major role in the seasonality of respiratory diseases, especially in the northern hemisphere where times of the year where humidity and temperature are the lowest coincide with winter holidays (Neumann and Kawaoka 2022).

The impact of seasonality on the transmission of respiratory diseases is often studied through epidemic models with sinusoidal transmission rates (Liu et al. 2010). However, a more realistic representation of the alternation between regular weeks and holidays is the two–state switch function adopted by Earn et al. (2000), where the transmission rate is assumed to be reduced during the summer time. Here, we assess the effect of seasonality as the result of two contrasting phenomena: on one hand, during the summer we assume that the transmission rates are reduced, which is beneficial from an epidemiological perspective; on the other hand, we consider also a reduction of the vaccination rates, which is instead detrimental.

Hence, following Buonomo et al. (2022), seasonality is incorporated in the transmission rates $a_i$ ($i=1,2,3$), the co–infection rate $a_4$ and the vaccination rates $\varphi _j$ ($j = 1,2$), by replacing the aforementioned parameters with the following ones:

$$\begin{aligned} a_{i,s}(t)&= \chi _i(t)\, a_i(\mathcal {V}(t)), &  i = 1, \dots , 4, \\ \varphi _{j,s}(t)&= \chi _{4+j}(t)\,\varphi _{j}(\mathcal {M}_j(t))), &  j = 1,2, \end{aligned}$$

where the subscript “*s*" means that the parameter under consideration incorporates seasonality and $\chi _l(t)$ ($l = 1, \dots , 6$) is defined to be the two–states switch representing the percentage of reduction $\epsilon _l$ in the *l*–th rate occurring during the summer season:

$$\begin{aligned} \chi _l (t) = {\left\{ \begin{array}{ll} 1 & \text {if } 0 \le t< 181 , \\ 1- \epsilon _l & \text {if } 181 \le t \le 243 , \\ 1 & \text {if } 243 < t \le 365 . \end{array}\right. } \end{aligned}$$

Assuming that the initial time $t_0$ corresponds to January 1, the functions $\chi _l$ repeat periodically at intervals of length 365 days. Day $t =181$ corresponds to July 1 and day $t =243$ corresponds to September 1.

We consider two scenarios, say *seasonality* (resp. *no–seasonality*), in which seasonality is included (resp. neglected) and set $\epsilon _l = 0.25$ ($l = 1, \dots , 6$). The other parameter values are as in Tables 2 and 3. Figure 8 shows the oscillating dynamics of the seasonality scenario (red solid lines) overlapped with the solutions obtained in the no–seasonality scenario (black dotted lines). In panel 8a, where these last converge to the co–endemic equilibrium, seasonality triggers sustained oscillations, as it could be expected. In panel 8b, sustained oscillations occurs in both scenarios. However, prevalence peaks are higher in the seasonal scenario, indicating that the detrimental effects due to the reduction of the vaccination rates prevail on the beneficial ones due to the reduction of transmission. We finally point out that corners are visible at $t_{1,k}= 181 + 365k$ and $t_{2,k} =243 + 365k$, $k \in \mathbb {N}$, due to the jump discontinuities in the vector field.

## Concluding remarks

The interplay between co–infections and human behaviour from a modelling perspective is attracting the attention of scholars (Hendrickx et al. 2019), although, to the best of our knowledge, it remains a topic that has been seldom explored in the literature. Here, we make new contributions by extending the qualitative analysis of the model and including behavioural changes through the use of the information index, which is a well-established method of the Behavioural Epidemiology of Infectious Diseases.

Firstly, we extend the compartmental co–infection model proposed in Fahlena et al. (2022) to a *behavioural* model by assuming that the contact rate depends on the information index. A qualitative analysis of the model is carried out through stability and bifurcation theory. We compute the control reproduction number $\mathcal {R}_C$, which depends on the percentage reduction of contact rate due to compliance with public health policies. The classical threshold condition, $\mathcal {R}_C = 1$, holds, with a branch of stable endemic equilibria emerging from $\mathcal {R}_C = 1$. In order to rule out the existence of both endemic and co–endemic equilibria when $\mathcal {R}_C < 1$, we determine the direction of the bifurcation at $\mathcal {R}_C = 1$, showing the existence of a forward transcritical bifurcation.

To analyse the local stability of the boundary endemic equilibria, we computed the invasion numbers. As for the existence and local stability of the coendemic equilibria, the determination of sufficient conditions for their existence and local stability is mathematically challenging, due to the complexity of the model. To overcome this problem, we use the center manifold theory to determine conditions that guarantee the existence of a transcritical forward bifurcation, deriving the local stability of the coendemic equilibria at least when the invasion number is close to the value 1. This result constitutes a novel contribution of this work, as to the best of our knowledge, the stability of co–endemic equilibria is usually not addressed in the literature for similar co–infection models.

In addition, through numerical simulations, we find that information–induced human behavioural changes can stabilise the system dynamics.

Secondly, the model is further extended to include vaccination. We employ three information indexes to model the interaction between the prevalence–induced vaccination and the vaccination–induced relaxation of social distancing. The main result is that the latter phenomenon may have a destabilising effect.

Finally, we assess the impact of seasonality by introducing a two–state switch function to represent the reduction of both vaccination and transmission rates during the summer season. We find that seasonality may induce the onset of sustained oscillations where the peaks of infected individuals are higher compared to the case without seasonality.

Our findings can be developed in several directions. The first open problem is to prove the existence of Hopf bifurcation for both the main model (6) and the extended model. Although the complexity of the models makes the analysis challenging, it may be possible, in principle, to use an approach similar to that used for models with comparable complexity like, e.g., in Chen et al. (2024); Zhang et al. (2020). We also plan to estimate the co–infection rates and the information–related parameters by applying the model to specific co–infection of respiratory viruses like, e.g., influenza and SARS–CoV–2, where at least partial datasets are available (Dadashi et al. 2021; Dao et al. 2021; Krumbein et al. 2023; Yan et al. 2023). Finally, a possible follow–up of this study is the introduction of actions by the public health system aimed at reducing both the health and the economic impacts of the diseases. Optimal control theory is currently one one of the most widely used ways for this purpose (Hao et al. 2024). When the control acts in a specific way on the contact rate, new phenomena like period doubling may occur (Sharbayta et al. 2022). This will be the subject of future investigations.

## Data Availability

Data sharing not applicable to this article as no datasets were generated or analysed during the current study.

## Appendix A. Existence of a positive invariant set

### Lemma 1

Assume that all components of the initial data $\textbf{x}_0$ of model (8) are strictly positive. Then, all the components of the solution $\textbf{x}(t)$ are strictly positive for any $t > t_0$.

### Proof

Let us denote by $[t_0, \eta )$ the maximal interval of existence of the solution, with $t_0 < \eta \le +\infty$. Consider:

43 $$\begin{aligned} t_1 := \text {sup} \{\, t \mid x(\tau ) > 0, \, \tau \in [t_0, t] \,\} . \end{aligned}$$

Obviously, $t_1 \in (t_0, \eta ]$. Equation (6a) can be rewritten as:

44 $$\begin{aligned} \dot{S}(t) = b(t) - a(t) S(t), \end{aligned}$$

where $b(t):= \mu + \rho _1 R_1(t) + \rho _2 R_2(t) + \rho _3 R_3(t)$, and $a(t):= \mu + a_1(t)I_1(t) + a_2(t)I_2(t) + a_3(t)I_3(t)$. Multiplying both sides by $\exp {\int _{t_0}^t a(\tau ) d\tau }$, equation (44) becomes:

$$\begin{aligned} \frac{d}{dt} \left\{ S(t) e^{\int _{t_0}^t a(\tau ) d \tau } \right\} = b(t) e^{\int _{t_0}^t a(\tau ) d \tau }. \end{aligned}$$

Integrating both sides from $t_0$ to $t_1$, we obtain:

$$\begin{aligned} S(t_1) e^{\int _{t_0}^{t_1} a(\tau ) d \tau } - S(t_0) = \int _{t_0}^{t_1} b(s) e^{\int _{t_0}^s a(\tau ) d \tau } ds, \end{aligned}$$

and so:

$$\begin{aligned} S(t_1) = S(t_0) e^{- \int _{t_0}^{t_1} a(\tau ) d \tau } + e^{- \int _{t_0}^{t_1} a(\tau ) d \tau } \int _{t_0}^{t_1} b(s) e^{\int _{t_0}^s a(\tau ) d \tau } ds > 0, \end{aligned}$$

since *b* is positive on $$[t_0, t_1)$$. The same argument can be applied to show that $$x_i(t_1) > 0$$ for $$i = 2, \dots , 8$$ and therefore $$x(t_1) > 0$$.

Now, we show that $t_1 = \eta$ and therefore $x(t) > 0$, for every $t > t_0$. Suppose by contradiction that $t_1 < \eta$. Since *x*(*t*) is continuous, there exists $t_2 > t_1$ such that $x(t) > 0$ on $[t_0, t_2]$. This leads to a contradiction, due to (43). $\square$

### Proof of Proposition 1

Since the closure of a positively invariant set is still positively invariant (Amann 2011, Remark 16.3h), it follows from Lemma 1 that $\mathbb {R}_+^8$ is positively invariant.

To prove the upper bound on $x_8(t) = M(t)$, we start from equation (1). Since

45 $$\begin{aligned} \int _{0}^{+\infty } Erl_{1,a}(\tau ) \,d\tau = 1, \end{aligned}$$

we have that:

$$\begin{aligned} M(t)&= \int _{0}^{+\infty } g( I_1(t- \tau ) , \, I_2(t-\tau ), \, I_3(t-\tau ) ) \text {Erl}_{1,a}(\tau ) d\tau \\&\le \int _{0}^{+\infty } g(1, \, 1, \, 1) \text {Erl}_{1,a}(\tau ) d\tau = \sum _{i=1}^{3} k_i. \end{aligned}$$

The set $\Omega$ is then positively invariant for the system (8). Since the solution *x*(*t*) is confined in the compact set $\Omega$, it follows that $\eta = +\infty$ and the solution exists globally for $t > t_0$. $\square$

## Appendix B. Invasion criteria

In order to compute the invasion number $\mathcal {R}_2^{\text {inv}}$ of virus–2 at the equilibrium $E_1$, we introduce:

$$\begin{aligned} \mathcal {F}_2 = { \begin{bmatrix} a_2(M)S I_2 + (1-p)a_3(M)SI_3 + a_2(M)R_1I_2 + a_3(M)R_1I_3\\ a_4(M)[I_1I_2 + I_1I_3 + I_2I_3] \end{bmatrix} } \end{aligned}$$

and

$$\begin{aligned} \mathcal {V}_2 = { \begin{bmatrix} (1-q)a_4(M)I_1I_2 + a_4(M)I_2I_3 +(\gamma _2+ \mu )I_2\\ (\gamma _3+ \mu )I_3 \end{bmatrix}. } \end{aligned}$$

The Jacobian matrices of $\mathcal {F}_2$ and $\mathcal {V}_2$ evaluated at the virus–1 dominance equilibrium $E_1$ give the transmission matrix

$$\begin{aligned} F_2= { \begin{bmatrix} \frac{a_2^*[(\mu + \rho _1)(\mu + \gamma _1) + \gamma _1a_1^*I_1^*]}{(\mu +\rho _1)a_1^*} & \qquad \frac{a_3^*[(1-p)(\mu + \rho _1)(\mu + \gamma _1)+ \gamma _1a_1^* I_1^*]}{(\mu + \rho _1)a_1^*} \\ a_4^*I_1^* & \qquad a_4^*I_1^* \end{bmatrix}, } \end{aligned}$$

and the transition matrix

$$\begin{aligned} V_2= { \begin{bmatrix} (1-q) a_4^*I_1^* + \gamma _2 +\mu & \qquad 0 \\ 0 & \qquad \gamma _3 + \mu \end{bmatrix}. } \end{aligned}$$

The invasion number $\mathcal {R}_2^{\text {inv}}$ is the spectral radius of the following invasion matrix (Van den Driessche and Watmough 2002):

$$\begin{aligned} \Pi = F_2V_2^{-1} = { \begin{bmatrix} \Pi _{11} & & \qquad \Pi _{12} \\ \Pi _{21} & & \qquad \Pi _{22} \end{bmatrix}. } \end{aligned}$$

### Proof of Proposition 4

Since $\mathcal {R}_{C1}>1$, the equilibrium $E_1$ exists and is unique (see Subsection 3.3). As for its local stability, conditions (A1)–(A4) of Vanden Driessche and Watmough (2002, Theorem 2) are trivially satisfied. Condition (A5) requires that if $\mathcal {F}_2$ is set to zero, then all eigenvalues of the Jacobian matrix $J(E_1)$ have negative real parts. In this case, four of the eigenvalues of $J(E_1)$ are $\lambda _1 = -(\rho _3 + \mu )$, $\lambda _2 = -(\gamma _2 + \mu )$, $\lambda _3 = -(\gamma _3 + \mu )$, $\lambda _4 = -(a_1^*I_1^*+ \rho _2 +\mu )$, while the remaining four eigenvalues can be determined from the 4x4 sub–matrix

46 $$\begin{aligned} \bar{J_1} = { \begin{bmatrix} -(\mu +a_1^*I_1^*)& -(\mu + \gamma _1) & \rho _1 & \frac{|a_1'(M_1^*)|}{a_1^*}(\mu +\gamma _1)I_1^*\\ a_1^*I_1^* & 0 & 0 & - \frac{|a_1'(M_1^*)|}{a_1^*}(\mu +\gamma _1)I_1^* \\ 0 & \gamma _1 & -(\rho _1+\mu ) & 0 \\ 0 & \frac{k_1}{T_a} & 0 & -\frac{1}{T_a} \end{bmatrix}, } \end{aligned}$$

whose characteristic polynomial is:

47 $$\begin{aligned} P(\lambda ) = (\lambda + \mu )[(\lambda + q)(\lambda ^2 + l_1\lambda + l_2)+( m_1\lambda + m_2)], \end{aligned}$$

where:

$$\begin{aligned} &  m_1&= \gamma _1a_1^*I_1^*,&l_1&= \frac{1}{T_a} + a_1^*I_1^*, &  \\ &  m_2&=\frac{\gamma _1a_1^*I_1^*}{T_a},&l_2&= \frac{a_1^*I_1^*}{T_a} + \frac{k_1|a_1'(M_1^*)|}{T_aa_1^*}, &  \\ &  q&= \mu + \rho _1.&\end{aligned}$$

Then, another eigenvalue is $\lambda _5=-\mu$, the others are the roots of the equation:

$$\begin{aligned} \lambda ^3 + \mathcal {A}_1\lambda ^2 + \mathcal {A}_2 \lambda + \mathcal {A}_3 =0, \end{aligned}$$

with $$\mathcal {A}_1 = l_1 + q$$, $$\mathcal {A}_2 = l_2 + m_1 + ql_1$$ and $$\mathcal {A}_3 = ql_2 + m_2$$.

Routh–Hurwitz criterion states that all roots are negative or have a negative real part if and only if: $(i)\,\mathcal {A}_i > 0$, $i=1,2,3$, $(ii)\,\mathcal {A}_1 \mathcal {A}_2 > \mathcal {A}_3$. The condition (*i*) is clearly satisfied, while as for condition (*ii*) is enough to observe that:

$$\begin{aligned} \mathcal {A}_1 \mathcal {A}_2= &  (l_1+q)(l_2 + m_1 + ql_1)> \left( \frac{1}{T_a} + q \right) (l_2 + m_1) > ql_2 + \frac{m_1}{T_a}\\= &  ql_2 + m_2 = \mathcal {A}_3. \end{aligned}$$

Hence, the eigenvalues of $J(E_1)$ have negative real part and the condition (A5) of Van den Driessche and Watmough (2002, Theorem 2) is satisfied. $\square$

## Appendix C. Balance equations for the extended model

The model developed in Section 5 is described by the following system of non–linear ordinary differential equations:

48a $$\begin{aligned} \dot{S}(t)&= \mu + \sum _{i=1}^3 [\rho _iR_i + \theta _iV_i - a_iSI_i] - (\mu + \varphi _1 + \varphi _2)S,\end{aligned}$$

48b $$\begin{aligned} \dot{I_1}(t)&= a_1(S + R_2 + \sigma _1 V_1 + V_2 + \sigma _1 V_3)I_1 \nonumber \\&\quad + a_3[p(S + \sigma _1V_1 + V_2 + \sigma _1V_3) + R_2 ]I_3\nonumber \\&\quad - a_4(qI_1I_2 + I_1 I_3) - (\gamma _1 + \mu )I_1,\end{aligned}$$

48c $$\begin{aligned} \dot{I}_2(t)&= a_2( S + R_1 +V_1 + \sigma _2 V_2 + \sigma _2 V_3)I_2 \nonumber \\&\quad + a_3[(1-p)(S + V_1 + \sigma _2V_2 + \sigma _2V_3)+ R_1]I_3\end{aligned}$$

48d $$\begin{aligned}&\quad - a_4[(1-q)I_1I_2 + I_2 I_3] - (\gamma _2 + \mu )I_2,\end{aligned}$$

48e $$\begin{aligned} \dot{I}_3(t)&= a_4(I_1I_2 + I_1I_3 + I_2I_3) - (\gamma _3 + \mu )I_3, \end{aligned}$$

48f $$\begin{aligned} \dot{R}_1(t)&= \gamma _1I_1 - (a_2I_2 + a_3I_3)R_1 - (\rho _1 + \mu ) R_1,\end{aligned}$$

48g $$\begin{aligned} \dot{R}_2(t)&= \gamma _2I_2 - (a_1I_1 + a_3I_3)R_2 - (\rho _2 + \mu )R_2,\end{aligned}$$

48h $$\begin{aligned} \dot{R}_3(t)&= \gamma _3I_3 - (\rho _3 + \mu )R_3, \end{aligned}$$

48i $$\begin{aligned} \dot{V}_1(t)&= \varphi _1S - [\sigma _1a_1I_1 + \sigma _1pa_3I_3 + a_2I_2 + (1-p)a_3I_3]V_1 \nonumber \\&\quad - (\varphi _2 + \theta _1 + \mu )V_1,\end{aligned}$$

48j $$\begin{aligned} \dot{V}_2(t)&= \varphi _2S - [a_1I_1 + pa_3I_3 + \sigma _2 a_2I_2 + \sigma _2 (1-p)a_3I_3]V_2 \nonumber \\&\quad - (\varphi _1+ \theta _2 + \mu )V_2,\end{aligned}$$

48k $$\begin{aligned} \dot{V}_3(t)&= \varphi _2V_1 + \varphi _1V_2 - [\sigma _1 a_1I_1 + \sigma _1 pa_3I_3 + \sigma _2 a_2I_2 \nonumber \\&\quad + \sigma _2 (1-p)a_3I_3]V_3 - (\mu + \theta _3) V_3,\end{aligned}$$

48l $$\begin{aligned} \dot{\mathcal {M}}_1(t)&= \frac{1}{T_1}\big ( g_1(I_1,I_3)- \mathcal {M}_1 \big ),\end{aligned}$$

48m $$\begin{aligned} \dot{\mathcal {M}}_2(t)&= \frac{1}{T_2}\big ( g_2(I_2,I_3)- \mathcal {M}_2 \big ),\end{aligned}$$

48n $$\begin{aligned} \dot{\mathcal {V}}(t)&= \frac{1}{T_v}\big ( h(V_1, V_2, V_3)- \mathcal {V} \big ) . \end{aligned}$$

The rates $a_i$ ($i =1,2,3$) depend on the information index $\mathcal {V}$; the rate $\varphi _j$ depends on the information index $\mathcal {M}_j$ ($j=1,2$). The explicit dependence is here omitted for the sake of clarity. The system is equipped with the following initial conditions:

49 $$\begin{aligned} \begin{aligned} S(t_0) > 0, I_i(t_0) \ge 0 , \quad R_i(t_0) \ge 0, \quad V_i(t_0) \ge 0, \quad i =1,2,3, \\ \mathcal {M}_1(t_0) \ge 0 , \quad \mathcal {M}_2(t_0) \ge 0 , \quad \mathcal {V}(t_0) \ge 0 . \end{aligned} \end{aligned}$$

We also set $N(t_0) = 1$, so that $N(t) = 1$ for $t > t_0$.

## Appendix D. Basic analysis for the extended model

Using the same arguments of Proposition 1, we can show that the solutions of the extended model exist, are unique in the interval $[t_0, \infty )$ and are contained in the following positively invariant set $\mathcal {D}$:

$$\begin{aligned} {\mathcal {D} = \left\{ \textbf{x} \in \mathbb {R}^{13}_+ \ : \ \sum _{i=1}^{10} x_i =1, \ \mathcal {M}_{1} \le k_1 + k_3,\ \mathcal {M}_{2} \le k_2 + k_3, \ \mathcal {V} \le \sum _{i = 1}^3 \tilde{k}_i \right\} .} \end{aligned}$$

The extended model admits the disease–free equilibrium:

$$\begin{aligned} E^* = (S^*, 0, 0, 0, 0, 0, 0, V_1^*, V_2^*, V_3^*, 0, 0, \mathcal {V}^*), \end{aligned}$$

where:

$$\begin{aligned} S^*&= \frac{[\eta _1 - (1 + \rho _2) \varphi _{0,1}][\eta _2 - (1 + \rho _1) \varphi _{0,2}]}{\eta _1\eta _2 - \varphi _{0,1}\varphi _{0,2}(1+ \rho _1)(1+ \rho _2)},&V_3^*&= \rho _2V_1^* + \rho _1 V_2^* , \\ V_i^*&= \varphi _{0,i}\frac{\eta _j-(1 + \rho _i)\varphi _{0,j}}{\eta _1\eta _2 - \varphi _{0,1}\varphi _{0,2}(1+ \rho _1)(1+ \rho _2)},&\mathcal {V}^*&= \tilde{k}_1 V_1^* + \tilde{k}_2 V_2^* + \tilde{k}_3 V_3^*, \end{aligned}$$

and

$$\begin{aligned} \rho _i = \frac{\varphi _{0,i}}{\mu + \theta _3}, \qquad \eta _i = \varphi _{0,1} + \varphi _{0,2} + \theta _i + \mu + \rho _j \varphi _{0,i}, \end{aligned}$$

for $$i =1,2$$ and $$j \ne i$$.

The basic reproduction number $\mathcal {P}_0$ of the extended model coincides with the one obtained for model (6). As for the control reproduction number $\mathcal {P}_c$, we follow the notations adopted by Diekmann et al. (1990) and Van den Driessche and Watmough (2002) and introduce:

$$\begin{aligned} \mathcal {F} = {\displaystyle { \begin{bmatrix} a_1(\mathcal {V})(S + R_2 + \sigma _1V_1 + V_2 + \sigma _1V_3)I_1 + a_3(\mathcal {V})\\ [p(S + \sigma _1V_1 + V_2 + \sigma _1V_3) + R_2]I_3 \\ a_2(\mathcal {V})(S + R_1 + V_1 + \sigma _2V_2 + \sigma _2V_3)I_2 + a_3(\mathcal {V})\\ [(1-p)(S + V_1 + \sigma _1V_2 + \sigma _2V_3) + R_1]I_3 \\ a_4(\mathcal {V})(I_1I_2 + I_1I_3 + I_2I_3) \end{bmatrix} } } \end{aligned}$$

and

$$\begin{aligned} \mathcal {V} = { \begin{bmatrix} a_4(\mathcal {V})(qI_1I_2 + I_1 I_3) + (\gamma _1+ \mu )I_1\\ a_4(\mathcal {V})[(1-q)I_1I_2 + I_2 I_3] + (\gamma _2+ \mu )I_2\\ (\gamma _3+ \mu )I_3 \end{bmatrix}.} \end{aligned}$$

The transmission matrix *F* is given by:

$$\begin{aligned} F = { \begin{bmatrix} a_1(\mathcal {V}^*)\omega _1 & \qquad 0 & \qquad pa_3(\mathcal {V}^*)\omega _1\\ 0 & \qquad a_2(\mathcal {V})\omega _2 & \qquad (1-p)a_3(\mathcal {V})\omega _2\\ 0 & \qquad 0 & \qquad 0 \end{bmatrix},} \end{aligned}$$

where $$\omega _i = 1-(1-\sigma _i)(V_i^* + V_3^*)$$, $$i=1,2$$.

The transition matrix *V* is given by:

$$\begin{aligned} V = { \begin{bmatrix} \mu + \gamma _1 & \qquad 0 & \qquad 0\\ 0 & \qquad \mu + \gamma _2 & \qquad 0\\ 0 & \qquad 0 & \qquad \mu + \gamma _3 \end{bmatrix}.} \end{aligned}$$

The control reproduction number $\mathcal {P}_c$ is the spectral radius of the next generation matrix $K = FV^{-1}$. We have:

$$\begin{aligned} K = { \begin{bmatrix} \frac{a_1(\mathcal {V}^*)\omega _1}{\mu + \gamma _1} & \qquad 0 & \qquad pa_3(\mathcal {V}^*)\omega _1\\ 0 & \qquad \frac{a_2(\mathcal {V}^*)\omega _2}{\mu + \gamma _2} & \qquad (1-p)a_3(\mathcal {V}^*)\omega _2\\ 0 & \qquad 0 & \qquad 0 \end{bmatrix}.} \end{aligned}$$

By introducing ${\mathcal {P}_{c}}_i:= K_{ii}$ ($i=1,2$) and using equation (38) and equation (39), we get:

50 $$\begin{aligned} {\mathcal {P}_{c}}_i&= \left( 1 - \frac{ c_0 }{\hat{c}(1 + \tilde{D} \mathcal {V^*})}\right) \omega _i{\mathcal {R}_{0}}_i . \end{aligned}$$

Finally, we obtain the control reproduction number $\mathcal {P}_c$:

$$\begin{aligned} \mathcal {P}_c = \max \{{\mathcal {P}_{c}}_1, {\mathcal {P}_{c}}_2\}. \end{aligned}$$

From Van den Driessche and Watmough (2002, Theorem 2), we can derive the classical threshold condition:

### Proposition 6

The disease-free equilibrium $E^*$ of the extended model is locally asymptotically stable if $\mathcal {P}_c < 1$ and unstable if $\mathcal {P}_c > 1$.
